# Characterization of Inflammatory Mediators and Metabolome in Interstitial Fluid Collected with Dermal Open Flow Microperfusion before and at the End of Dupilumab Treatment in Atopic Dermatitis

**DOI:** 10.1021/acs.jproteome.4c00153

**Published:** 2024-07-10

**Authors:** Fernanda Monedeiro, Barbara Ehall, Katrin Tiffner, Anita Eberl, Eva Svehlikova, Barbara Prietl, Verena Pfeifer, Julia Senekowitsch, Anu Remm, Ana Rebane, Christoph Magnes, Thomas Pieber, Frank Sinner, Thomas Birngruber

**Affiliations:** † HEALTH − Institute for Biomedical Research and Technologies, Joanneum Research Forschungsgesellschaft mbH, Neue Stiftingtalstraße 2, Graz 8010, Austria; ‡ Division of Endocrinology and Diabetology, Medical University of Graz, Neue Stiftingtalstraße 6, Graz 8010, Austria; § BioTechMed, Mozartgasse 12, Graz 8010, Austria; ∥ Center for Biomarker Research in Medicine (CBmed) GmbH, Stiftingtalstrasse 5, Graz 8010, Austria; ⊥ Institute of Biomedicine and Translational Medicine, University of Tartu, Biomeedikum, Ravila 19, Tartu 50411, Estonia

**Keywords:** interstitial fluid, metabolomics, cytokines, atopic dermatitis, dupilumab

## Abstract

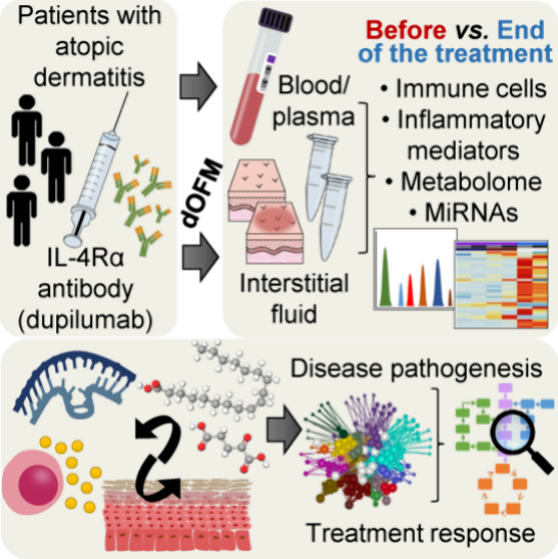

Dupilumab is a monoclonal antibody approved for the treatment of atopic dermatitis (AD); however, its effects on molecular, cellular, and immunological levels remain to be elucidated. In this study, blood and dermal interstitial fluid (ISF) from nonlesional (NL) and lesional (L) skin were collected from eight patients with moderate to severe AD, before (visit 2-v2) and at the end of a 16-week treatment with dupilumab (visit 10-v10). Clinical treatment effect was demonstrated by significantly decreased AD severity scores at the end of treatment. At v10 versus v2, the percentages of CD4+ interleukin-producing cells showed a decreasing trend in ISF L and NL, unbound IL-4 levels in plasma were increased, IL-5 levels in ISF L reduced, and levels of factors involved in anti-inflammatory pathways and re-epithelization increased. At v2, ISF L showed that AD lesions might have altered amino acid pathways and lipid signaling compared to ISF NL. At v10, ISF L exhibited raised levels of long- and very-long-chain fatty acids and lipids compared to v2. Furthermore, dupilumab administration caused reduced expression of miR-155–5p and miR-378a-3p in ISF L. In conclusion, results from the present study provided novel knowledge by linking local immune and metabolic alterations to AD pathogenesis and treatment response.

## 1 Introduction

Atopic dermatitis (AD) is a chronic and relapsing inflammatory disease that causes severe itching and eczematous lesions. AD is characterized by exaggerated T helper 2 (Th2) cell-mediated immune responses favoring skin disruption.^1^ The Th2 cytokines IL-4 and IL-13 are well-known as key drivers of this disease, therefore posing as targets of recent therapeutic advances. Dupilumab is a recombinant human monoclonal antibody of immunoglobulin G4 that binds to the shared alpha subunit of the IL-4 receptor (IL-4Rα), thus inhibiting IL-4 and IL-13 signaling. Currently, dupilumab is FDA-approved for the treatment of moderate to severe AD.^2^ Despite dupilumab’s proven clinical efficacy, the underlying molecular, cellular, and immunological changes in epidermal pathology remain to be elucidated. Moreover, immune activation extends beyond lesional AD because nonlesional skin and blood components may also reflect mechanisms of AD pathogenesis, possibly expressing AD-specific molecular changes.

The innovative potential of the present investigation relies on the application of the sampling technology dermal open flow microperfusion (dOFM) that provides the unique possibility to collect diluted but otherwise unchanged dermal interstitial fluid (ISF) due to the open mesh design of the probes.^3,4^ Dermal ISF, which is directly and in a minimally invasive manner sampled with dOFM in lesional and nonlesional skin, contains inflammatory biomarkers and metabolites as well as whole immune cells.^5−7^ Other methods to collect skin-related samples such as skin biopsies and suction blisters are more invasive than dOFM, whereas tape stripping, being less invasive than dOFM, is limited to samples from the superficial part of the dermis, the *stratum corneum*. In contrast to these mentioned sampling techniques, dOFM provides direct access to unchanged dermal ISF, enabling a deep insight into tissue processes *in vivo*, and thus representing a method that monitors *in vivo* scenarios as close as possible to address the correlation between biomarkers and clinical effects of dupilumab.

So far, dupilumab effects on AD molecular and immunological profiles have been mostly explored in blood samples or skin biopsies.^8,9^ Hamilton et al. have profiled gene expression in the skin of patients treated with dupilumab, demonstrating for the first time that anti-IL-4Rα therapy results in the alteration of molecular signatures related to AD pathogenesis.^8^ The analysis of metabolites (particularly lipids) from AD serum and *stratum corneum* has been previously used to assess the response to dupilumab treatment.^10,11^ However, an integration of tissue-specific metabolomics, immune profiles, and clinical outcome measurements remains inedited.

16-week dupilumab treatment along with the comprehensive analysis of immune cell populations, inflammatory mediators, microRNAs (miRNAs), and metabolome in dermal ISF from nonlesional (NL) and lesional (L) skin as well as in whole blood and plasma samples was performed. In this way, this study provides an unprecedented holistic view of dupilumab effects considering different cellular and molecular levels.

## 2 Experimental Section

### 2.1 Study Design and Dupilumab Treatment

This was a single center, noncontrolled, open exploratory clinical study (Eudra CT Number: 2018–003642–17; DRKS Number: DRKS00023872) conducted at the Center for Medical Research, Clinical Trial Unit at the Medical University of Graz. Written informed consent had been obtained from all participants before any study-related activities started. The clinical study was approved by the local ethics committee of the Medical University of Graz, Austria (31–030 ex 18/19) and performed in accordance with the ICH guidelines for Good Clinical Practice and the Declaration of Helsinki.^12,13^ We enrolled female and male AD patients at ages of 18 to 65 years (both inclusive) with chronic moderate to severe AD, diagnosed for at least six months prior to inclusion, classified by Eczema Area and Severity Index (EASI) score above 16 and Investigator’s Global Assessment (IGA) score greater than or equal to three, and not adequately controlled by topical medications. Also, participants were required to have at least one suitable AD lesion accessible for dOFM investigation. Main exclusion criteria were significant allergies to humanized monoclonal antibodies, known hypersensitivity to dupilumab or any of its excipients, pregnant and breastfeeding women, and women unwilling to use reliable contraception during the study. Detailed study inclusion and exclusion criteria are available in the Table S1.

**Figure 1:**
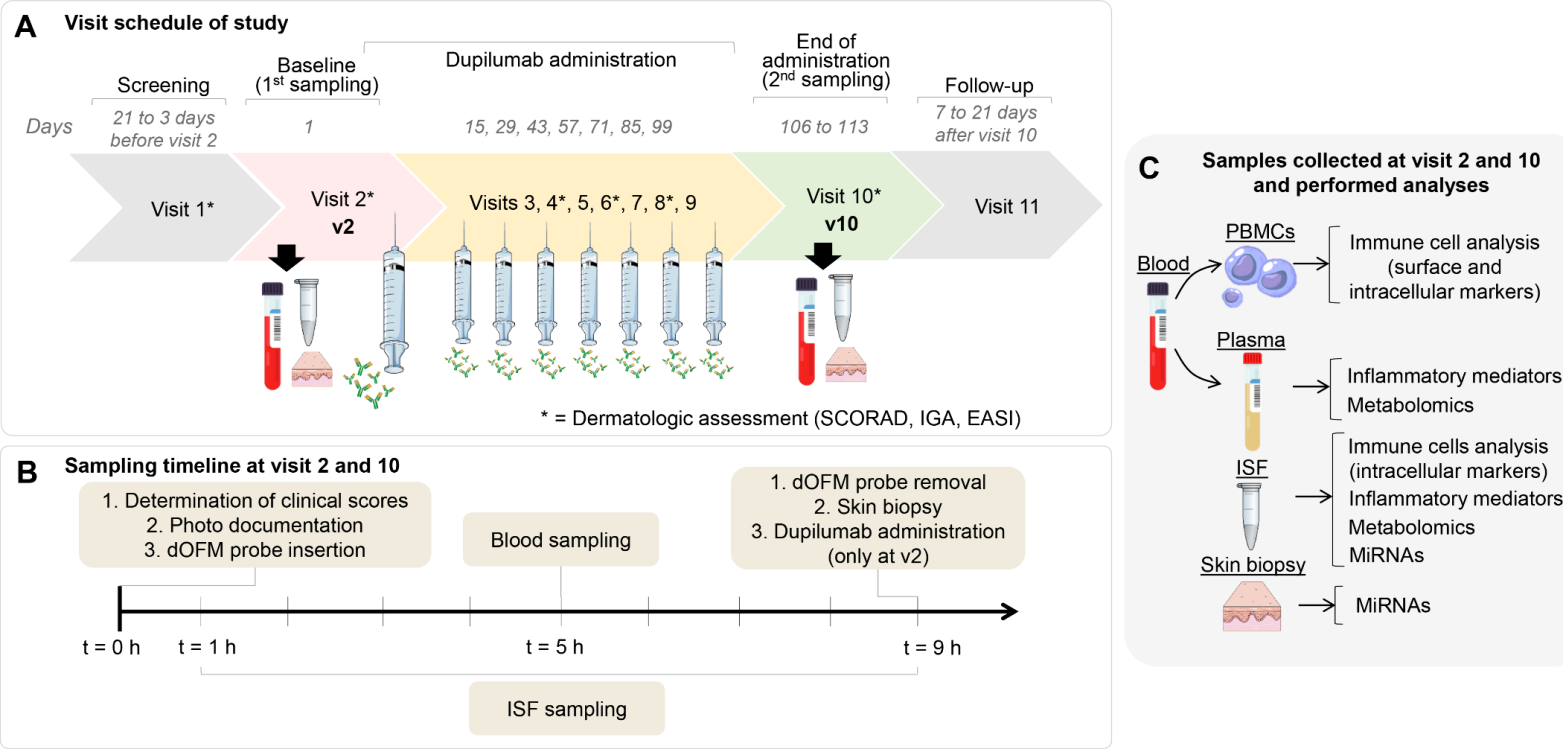
Study overview. (A) Visit schedule of the study; (B) sampling timeline at visits 2 and 10; (C) overview of samples collected at visits 2 and 10 and performed analyses.

Eight participants (5 female and 3 male) were enrolled in the study and attended 11 visits (Figure 1A). At visit 1 (v1, screening visit), participants’ eligibility was assessed, their dermatologic conditions were examined, and AD was rated using the clinical scores EASI, IGA, and Scoring Atopic Dermatitis (SCORAD).

Visit 2 (v2, baseline visit) took place on day 1 of the study. IGA, EASI, and SCORAD scores were recorded (Figure 1B) and dOFM sampling was performed for 8 h with bihourly collection of ISF samples. At 5 h, two blood samples were taken. After completion of dOFM sampling, skin biopsy was performed, and then 600 mg of dupilumab (initial dose) was administered subcutaneously according to the standard treatment regimen. At visits 3 to 9 (at the days 15, 29, 43, 57, 71, 85, and 99), each participant received 300 mg dupilumab subcutaneously (total 16 weeks). EASI, IGA, and SCORAD were assessed prior to every second dupilumab administration (at visits 4, 6, and 8). Visit 10 (v10, end of treatment visit) was scheduled 7–13 days after visit 9, and in v10, the procedures of v2 were repeated (except for dupilumab administration). Visit 11 (v11, follow-up visit) was scheduled 7 to 21 days after v10. The samples collected at v2 and v10 underwent several analyses (Figure 1C): Blood samples were analyzed for immune cells and inflammatory mediators; ISF samples were analyzed for immune cells, inflammatory mediators, metabolome, and miRNA composition. Skin biopsy samples were analyzed for their miRNAs composition.

### 2.2 dOFM Sampling and Processing of ISF Samples

dOFM sampling was performed at v2 and v10. Prior to dOFM probe insertion, the location, size, and conditions of both lesional (L) and nonlesional (NL) skin sites were documented by placing a mark in a schematic human outline and by taking a picture of each site (*t* = 0). In each participant, four dOFM probes (DEA15003, OD 0.5 mm, 30 mm open-mesh, CE-certified for human use, Joanneum Research GmbH, Graz, Austria) were placed each in L and NL skin at defined sites after appropriate disinfection. The fundamental premise was to use the same locations in v2 and v10. When after treatment with dupilumab the participants did not show any lesions anymore (which was mostly the case), the same sites were selected in v10 as in v2; i.e., four dOFM probes in NL skin and four in the former L skin. However, if at v10 lesions were present at a different site than in v2, the dOFM probes were placed in these lesions in v10. After dOFM probe insertion, the puncture sites were sealed with a medical adhesive.

Three of the probes each at L and NL sites were perfused with albumin-containing perfusate (ELO-MEL isoton with 2% human serum albumin, Fresenius Kabi, Graz, Austria) and one with albumin-free perfusate (ELO-MEL isoton, Fresenius Kabi, Graz, Austria). Afterward, the probes were connected to wearable dOFM pumps assuring the perfusion with a flow of 1 μL/min and dOFM sampling was started 1 h after the probe insertion was completed (at *t* = 1 h). Sampling of dermal ISF was performed for 8 h until *t* = 9 h and ISF was collected every 2 h (Figure 1B). Further details regarding the dOFM setup can be found elsewhere.^3,5,14−16^ After the sampling was completed, the dOFM probes were removed, and the collected ISF samples were stored at −80 °C until analysis.

The collected ISF samples from the dOFM probes perfused with albumin-containing perfusate were centrifuged (5 min, 400*g*, RT), and the supernatant was used for analysis of inflammatory mediators and miRNA composition. The sediment was subjected to immune cell analysis. ISF samples from the dOFM probes with albumin-free perfusate were utilized for metabolomics.

### 2.3 Blood Samples and Skin Biopsies

Blood samples were collected each at *t* = 5 h at v2 and v10 in a K3EDTA tube (Vacuette, tripotassium ethylenediaminetetra-acetic acid, 2 mL, Greiner, Kremsmünster, Austria) and in a lithium heparin tube (Vacuette, 8 mL, Greiner, Kremsmünster, Austria). The blood samples collected in the K3EDTA tube were centrifuged (5 min, 400*g*, RT) and the generated plasma samples were kept at −80 °C until further analysis. The blood samples collected in the lithium heparin tube were used for the isolation of peripheral blood mononuclear cells (PBMCs).

#### 2.3.2 PBMC Isolation for Deep Immune Phenotyping

Whole blood (100 μL) was used directly for surface staining. The remaining heparinized blood was diluted 1:1 (v/v) with phosphate-buffered saline (PBS, Fisher Scientific, Vienna, Austria) and layered into a tube prefilled with lymphoprep density gradient media (Stemcell Technologies, Cologne, Germany). Then, density gradient centrifugation was performed (20 min, 800*g*, RT) and PBMCs were collected and immediately washed with PBS. Viability and number of the cells were assessed using an automated dual fluorescence cell counter (LUNA-FL, Logos Biosystems, Villeneuve d’Ascq, France), and then the cells were further cultivated for the measurement of cytokine production.

#### 2.3.3 Skin Biopsies

Skin punch biopsy samples (diameter: 2 m, sample weight: 9 to 10 mg) were taken from L skin after dOFM sampling was completed before dupilumab administration both at v2 and at v10. The same biopsy site was used at v2 and v10. Each skin sample was placed in a tube filled with RNAlater solution (Thermo Scientific, Waltham, MA, USA) and flash frozen in liquid nitrogen immediately after extraction. Then, samples were stored at −80 °C until further analyses.

### 2.4 Analysis of Cytokines, Chemokines, and Eicosanoids

#### 2.4.1 Cytokines and Chemokines

The cytokines and chemokines IL-10, IL-13, IL-4, IL-5, IL-17A, IL-21, IL-22, IL-23, IL-27, IL-31, and MIP-3α were analyzed with two different kits (V-PLEX Th17 Panel 1 Human Kit and U-PLEX Human Biomarker Kit, both from Meso Scale Diagnostics, Rockville, MD, USA). All assays were performed according to the instructions of the manufacturer. Plate reads were employed with a Meso Scale Discovery Quickplex SQ 120 instrument (Meso ScaleDiscovery, Rockville, MD, USA). Quantification was performed by using Meso Scale Discovery Workbench software (Meso Scale Discovery). Additionally, cytokine screening was performed based on proximity extension assay, using an Olink Target 48 Cytokine panel (Olink Proteomics, Uppsala, Sweden), which comprised the quantification of CCL11, CCL13, CCL19, CCL2, CCL3, CCL4, CCL7, CCL8, CSF1,CSF2,CSF3,CXCL10, CXCL11, CXCL12, CXCL8, CXCL9, EGF, FLT3LG, HGF, IFNG, IL-10, IL-13, IL-15, IL-17A, IL-17C, IL-17F, IL-18, IL-1β, IL-2, IL-27, IL-33, IL-4, IL-6, IL-7, LTA, MMP1, MMP12, OLR1, OSM, TGFA, TNF, TNFSF10, TNFSF12, TSLP, and VEGFA. Data were retrieved in agreement with the Olink standard analysis protocol. A 4PL-curve was used to calculate the concentration, referring to the measured Normalized Protein eXpression values in samples analyzed in each run.

#### 2.4.2 Eicosanoids

The eicosanoids PGE2, 15-HETE, 12-HETE, 12-HEPE, 13-HODE, and 17-HDHA were analyzed by liquid chromatography with tandem mass spectrometry (LC-MS/MS), using a previously developed and validated method.^6^

### 2.5 Metabolomics

Sample extraction was done by means of the cold methanol method,^17^ using 90 μL of ISF or plasma samples as sample volume. Analysis was performed using a Vanquish ultrahigh-performance liquid chromatography (UHPLC) system (Thermo Fisher Scientific, Waltham, MA, USA) equipped with a NH_2_–Luna hydrophilic interaction column (2 × 150 mm, 3 μm; from Phenomenex, Torrance, CA, USA) and coupled with a QExactive mass spectrometer (Thermo Fisher Scientific). The injection volume was 10 μL, and samples were analyzed in positive and negative ionization modes. Blank samples (30% methanol and 70% water), UltimateMix (65% EDTA human plasma and 35% serum, used as a positive control for a broad range of metabolites), quality control samples (pooled after sample workup), and study samples were measured in a stratified randomized sequence. Samples were divided into two batches and thawed to room temperature before measurements. All batches were measured in one run. Raw data were converted into .mzXML using msConvert (ProteoWizard Toolkit v3.0.5). PeakScout was used to identify detected *m*/*z* of known metabolites based on a reference list containing respective mass and retention times.

### 2.6 Deep Immunophenotyping Using Flow Cytometry

#### 2.6.1 Immune Phenotyping via Surface Antigens

Surface antigens of the isolated PBMCs were stained with BD Lyse/Fix buffer (Becton Dickinson, Franklin Lakes, NJ, USA) according to the manufacturer’s instructions. All used antibodies for identifying immune cell phenotypes were purchased from ThermoFisher (Waltham, MA, USA) and Becton Dickinson (Franklin Lakes, MA, USA) and are listed in the Supporting Information (Table S2).

#### 2.6.2 Measurement of Stimulated Cytokine Production in Immune Cells

For assessment of cellular cytokine production, the isolated PBMCs (2 × 10^5^ cells per patient) and 100 μL of ISF samples from both L and NL skin were transferred to a 96-well cell culture plate (U-bottom, Greiner, Kremsmünster, Austria) with Roswell Park Memorial Institute (RPMI) 1640 media (including 10% FBS, l-glutamine, and penicillin/streptomycin, ThermoFisher). The cells were incubated for 4 h at 37 °C (5% CO_2_) with cell stimulation cocktail (ThermoFisher) and protein transport inhibitor cocktail (ThermoFisher) according to the manufacturer’s instructions. After incubation, the intracellular staining was performed using BD Pharmingen Transcription Factor Buffer according to the manufacturer’s instructions (Becton Dickinson). All used antibodies for surface and intracellular staining are listed in the Supporting Information (Table S3).

#### 2.6.3 Acquisition, Controls, and Analysis

All samples were acquired on a standardized four-laser FACS Fortessa SORP instrument (Becton Dickinson) and data was analyzed using the provided DIVA software (both by Becton Dickinson and Franklin Lakes, NJ, USA). UltraComp eBeads (ThermoFisher) were used for compensation, and fluorescence-minus-one controls were acquired for appropriate gating.

### 2.7 Measurement of miRNAs

#### 2.7.1 From Skin Biopsies

For RNA extraction, the samples were placed into MagNA Lyser tubes (Roche, Mannheim, Germany) with 700 μL of QIAzol lysis reagent (Qiagen, Hilden, Germany) and 1 μL of the RNA spike-in mix (UniSp2, UniSp4 and UniSp5). Then, the tubes were placed in the MagNA Lyser instrument (Roche, Mannheim, Germany) at 6500 rpm for 35 s, and 3 runs were performed. After cell disruption, samples were mixed with 200 μL of chloroform, vortexed, and centrifuged (15 min, 12,000*g*, 4 °C). The resulting supernatant was processed using the RNeasy Mini kit (Qiagen, Hilden, Germany). DNA digestion was performed using an iScript kit (Bio-Rad Laboratories, Singapore, Cat. No. 172–5035). Afterward, 2 μL of DNase Mastermix was added to the samples (iScript DNase + iScript DNase Buffer). Next, PCR strips were inserted in a T100 Thermal Cycler (Bio-Rad Laboratories, Singapore). A UniSp6 spike-in mix was used to monitor cDNA synthesis. Reverse transcription was performed using the miRCURY LNA RT kit by Qiagen, following manufacturer’s instructions. A 1:60 dilution with nuclease free water was prepared for each generated cDNA sample. Then, 3 μL of each cDNA sample was added to a 385-well plate. RT-qPCR was performed using a CFX device C1000 Touch Thermal Cycler (Bio-Rad, Hercules, CA, USA). Mean efficiency-corrected Cq values of the housekeeping genes (U6 and SNORD48) were used to normalize and further calculate the 2^–ΔΔCq^ and relative expression of each investigated miRNA. The following miRNAs were successfully evaluated in skin biopsies: miR-143–3p, miR-17–5p, miR-223–3p, miR-378a-3p, miR-146a-5p, and miR-155–5p.

#### 2.7.2 From ISF Samples

A total of 100 μL of freshly collected ISF (dOFM perfusate: EloMel with 2% HSA) was suspended in 300 μL of Qiazol (Qiagen, Hilden, Germany) and then stored at −80 °C until RNA purification. For RNA purification, 0.8 μg of MS2 carrier RNA (Merck, Darmstadt, Germany), 2.5 fmol Cel-miR-39 spike-in mimic (Qiagen, Hilden, Germany), and 400 μL of Qiazol were added, and the mixture was vortexed. Next, RNA was purified using the miRNeasy Mini kit according to the manufacturer’s instructions. MiRNA expression was analyzed by real-time PCR on a Quantstudio instrument (Life Technologies, Carlsbad, CA, USA). Total RNA samples (10 ng) were reverse-transcribed using a TaqMan MiNA Reverse Transcription Kit (Applied Biosystems, Foster City, CA, USA). RT-PCR was performed in duplicate with 5 × HOT FIREPol Probe qPCR Mix Plus (ROX, Solis BioDyne, Tartu, Estonia) and the following Taqman miRNA assays (Cat. No. 4427975): cel-miR-39–3p (used as technical control for RNA purification), hsa-miR-203a-3p, hsa-miR-155–5p, hsa-miR-28–5p, hsa-miR-31–5p, hsa-miR-146a-5p, hsa-let-7a, and rno-miR-422b (= has-miR-378a-3p). Relative miRNA expression was calculated according to the 2^–ΔΔCq^ method and normalized to the expression of hsa-let-7a.

### 2.8 Data Analysis

Data preparation steps, data analysis, and visualization were conducted in an R environment (R v.4.2.1), using RStudio console (v. 2022.02.03, PBC, Boston, MA, USA).

#### 2.8.1 Data Preparation

Immune cell data expressed in terms of relative composition (%) were arcsine-transformed prior to the conduction of the statistical tests. Values for inflammatory mediators, which showed a response below the method′s lower limit of quantification, were assumed as equal to zero for the subsequent statistical analysis.

Quality of the metabolomics data was assessed by the following parameters: Difference from target mass, retention time standard deviation, percentage of nonexisting values in relation to all detected values, relative standard deviation in quality control samples, adequate peak shape, drift with progressing measurement time or daily batch, and blank load in quality control samples. Samples were also evaluated regarding the presence of analytical outliers according to the median deviation and principal component analysis (PCA). A further description of the data quality evaluation in metabolomics can be found elsewhere.^18^ Nonexisting values occurring due to some punctual technical ineffectiveness during data acquisition or processing (considered as displayed by metabolites present in at least 50% of all study samples and with a median above the median of total area) were imputed using the Random Forest method, applying “missForest” package (ntree = 500, mtry = 3). Other nonexisting values (displayed by metabolites absent in more than 50% of the total samples and with a median below the median total area) were associated with responses below the limit of detection. These nonexisting values were replaced by values corresponding to half of the minimum peak area obtained for the respective metabolite. Then, the filled-out data were submitted to natural logarithm transformation and normalization using probabilistic quotient normalization. The latter step was conducted using “KODAMA” R package, and it applied QC medians as reference. Prior to statistical analysis, metabolomics data was filtered for the removal of compounds addressed as xenobiotics such as tartaric acid, azelaic acid, saccharin sodium, and magnesium stearate.

#### 2.8.2 Statistical Analysis

Statistical tests to assess normality and to compare means were performed using R “stats” functions, “rstatix” package, and IBM SPSS Statistics version 23 (IBM Corp., Armonk, NY, USA). Comparisons between the AD severity scores recorded in the different study visits were performed using a paired *t*-test in the case of EASI and SCORAD and a Wilcoxon test in the case of IGA due to asymmetric distribution of data. To assess differences between the percentage of immune cells and levels of cytokines, chemokines, eicosanoids, and miRNAs obtained before (v2) and at the end of treatment (v10) paired tests were used. To compare the values for the aforementioned analytes measured in ISF NL, ISF L, PBMCs, and plasma, unpaired tests were employed. For these variables both nonparametric and parametric methods were evaluated. A *t*-test was chosen if data presented a normal distribution in one of the individual study subgroups to be compared; otherwise, nonparametric methods (Wilcoxon and Mann–Whitney tests) were applied (Tables S4–S6). Metabolite responses were assessed in terms of normality, but also regarding their homoscedasticity using Levene’s test (leveneTest function from “car” package). Based on the above-mentioned analysis, parametric (*t*-test) or nonparametric methods were used, designed for independent variables (i.e., for the comparison between ISF L and ISF NL) or paired samples (i.e., for the comparison between v2 and v10). Adjusted *p* values, obtained by applying the Benjamini–Hochberg procedure, were also assessed for all statistical comparisons. PCA was performed using scaled data, employing “factoextra” and “factoMineR” packages. Correlation analysis was performed using “Hmisc” package, and results were expressed in terms of Pearson correlation coefficients (*r*). Correlation analysis input consisted of paired data obtained for each AD patient at v2 and v10 per plasma, ISF L and ISF NL sample. Pathway enrichment analysis (PEA) was performed based on Kyoto Encyclopedia of Genes and Genomes database,^19^ employing hypergeometric method and using “FELLA” package. PEA input data were the KEGG ID of metabolites significantly correlated with inflammatory mediators in ISF L, and those found as discriminant (*p* < 0.05) in the comparisons concerning ISF NL versus ISF L or v2 versus v10. Heatmaps were generated using the “pheatmap” package, Sankey plots were built using “sankeyD3”, and Chord diagrams were prepared with “circlize”. The “EnhancedVolcano” package was used to build volcano plots. All other charts and boxplots were created using “ggplot2” functions.

## 3 Results and Discussion

### 3.1 Dupilumab Administration Significantly Improved AD Clinical Scores and Altered Associated Immunophenotype

**Figure 2:**
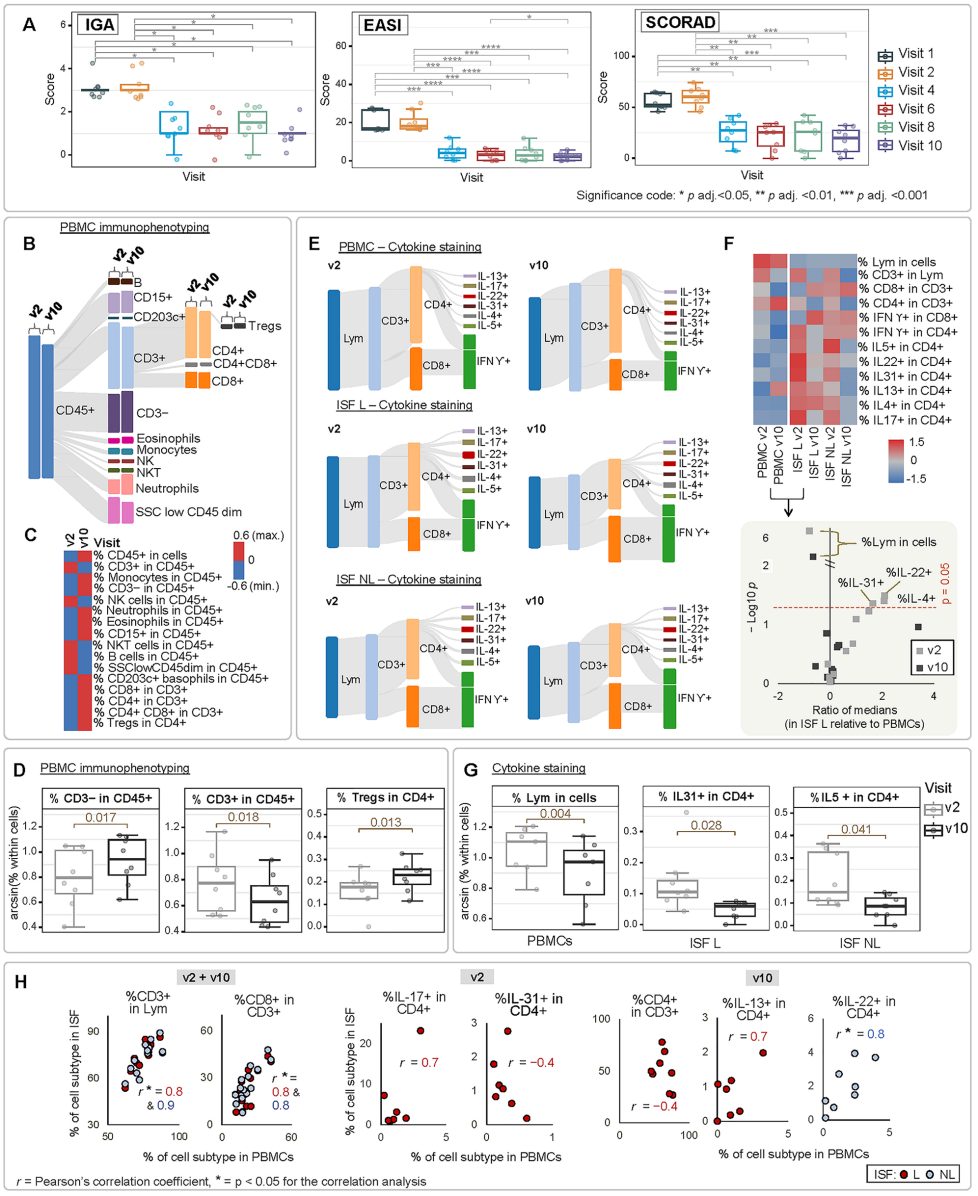
AD clinical scores and altered associated immunophenotype. (A) AD clinical scores recorded at the screening visit (v1), before (v2), during, and at the end (v10) of dupilumab treatment. (B) Flow cytometry panel of PBMC surface markers (PBMC immunophenotyping), expressed as Sankey diagrams (with bar heights reflecting cell proportion within the corresponding subtype) and (C) corresponding heatmap (scaled by cell subtype). (D) Percentage of immune cells significantly altered at v2 versus v10 for PBMC surface markers. (E) Flow cytometry panel of intracellular markers (cytokine staining), expressed as Sankey diagrams and (F) corresponding heatmap (scaled by cell subtype), along with the associated volcano plot showing differences for ISF L versus PBMCs. (G) Percentage of immune cells significantly altered at v2 versus v10 for intracellular markers. (H) Most relevant trends of immune cell populations in PBMCs versus ISF L and ISF NL. *P* adj. = *p* value adjusted according to the Benjamini–Hochberg procedure. Lym = lymphocytes.

Dermatologic assessment of AD severity was based on the EASI, IGA, and SCORAD scores. Decreased AD scores at the end of dupilumab treatment (visit 10–v10) compared to before the treatment (visit 2–v2) showed a substantial improvement of clinical status (*p* adj. < 0.05 for IGA; *p* adj. < 0.001 for both EASI and SCORAD) (Figure 2A). V2-derived AD scores were significantly decreased already at visit 4 (*p* adj. = 0.024 for IGA; *p* adj. = 0.0002 for EASI and *p* adj. = 0.002 for SCORAD). Overall, these results indicated that all participants responded well to the therapy, confirming dupilumab′s efficacy for the treatment of moderate to severe AD.

The analysis of peripheral blood mononuclear cell (PBMC) surface markers (PBMC immunophenotyping) showed that CD15+ and neutrophils (both in CD45+) were the main cell subtypes that increased at v10 versus v2, although in a statistically nonsignificant manner (Figure 2B,C). In contrast, dupilumab treatment led to a significant increase in CD3– (*p* = 0.017), together with a significant decrease in CD3+ cells (*p* = 0.018) (both in CD45+) (Figure 2D). Indeed, PBMCs from acute AD patients have been characterized by a higher percentage of CD3+ cells in relation to controls previously.^20^ Also, regulatory T cells, namely, Tregs (in CD4+), were significantly increased at v10 compared to v2 (*p* = 0.013) (Figure 2D). Tregs and Th17 cells function in a mutually antagonistic manner, with populations of Tregs being negatively correlated with Th17 in AD skin and blood.^21^ In this way, the lower number of Tregs prior to treatment may relate to more Th17 cells escaping immune inhibition, portraying a well-known aspect of the AD phenotype.^21^ Besides that, the increase in the number of Tregs at the end of the treatment may also indicate a decreased recruitment of Tregs to the AD skin site and/or reduced conversion of Tregs to Th2 cells as consequence of T cell plasticity influenced by the AD pro-inflammatory milieu.^22^

Regarding the panel of intracellular markers (cytokine staining) (Figure 2E,F), PBMCs exhibited a significant decrease in the percentage of lymphocytes at v10 (*p* = 0.004, Figure 2G), denoting amelioration of inflammation at the end of the treatment. Both PBMCs and ISF displayed a decrease of around 10% in the fraction of CD3+ within lymphocytes at v10 versus v2 (Figure 2E,F). AD skin immunophenotype is characterized by a greater infiltration of CD3+ lymphocytes, especially of the CD4+ subtype in comparison to the CD8+ subtype.^23^ In ISF samples, particularly in ISF L, the CD4+/CD8+ ratio was decreased at the end of the treatment (Figure 2E,F). Also in ISF (especially in ISF L), the proportion of IFN-Υ+ (in CD8+) was increased at the end of treatment (v10) compared to v2. Insufficient production of IFN-Υ appears to be an important aspect of innate immunity, playing an essential role in the pathogenesis of AD, and therefore, therapy with recombinant IFN-Υ has been considered for the correction of such immunological imbalances in AD.^24^ An opposite trend was observed in our study for PBMCs, where IFN-Υ+ percentages (in CD4+ and also in CD8+) were reduced at v10 versus v2.

Still regarding the assessed intracellular markers, dupilumab treatment also led to alterations in the interleukin populations within CD4+, which are highly relevant in the context of AD. At the end of the treatment (v10), the IL-22+ population (in CD4+) decreased compared to v2, particularly in ISF L (Figure 2E,F). IL-22 is believed to be a pathogenic cytokine in AD and potentially a driver of epidermal barrier defects.^25^ Furthermore, IL-22 blockade has shown clinical efficacy in patients with severe AD.^26^ In our study, the fraction of IL-31+ cells (in CD4+) was reduced in ISF at v10, and this change was significant in ISF L (*p* = 0.028) (Figure 2G). However, at v10, IL-31+ cells (in CD4+) remained increased in ISF L when compared to ISF NL (*p* = 0.043, the only statistically significant difference observed when comparing immune cells in ISF L versus ISF NL). These results may indicate attenuation of pruritus, which is driven by IL-31,^27^ while emphasizing the local role of this cytokine. In AD, eosinophils are more activated and sensitive to stimulus; in parallel, IL-5 has a pivotal role in eosinophil priming.^28^ Our results showed that the IL-5+ fraction (in CD4+) was reduced at v10 compared to v2 in ISF (and this reduction was significant in the case of ISF NL, *p* = 0.041, Figure 2G).

The median fraction of lymphocytes in cells was significantly different in the studied biological matrices, ranging from 68.3 (v2) to 78.9% (v10) in PBMCs, and from 2.5 to 7.8% in ISF (considering both visits). Nonetheless, the fraction of lymphocyte subtypes here evaluated across PBMCs and ISF did not show striking differences – with the exception of IL-4+, IL-22+, and IL31+ populations (in CD4+), which were significantly increased in ISF L when compared to PBMCs (volcano plot at Figure 2F). Although the literature on the immune cell landscape in ISF remains scarce, one possible explanation for such a similarity is the contribution of circulating blood to the ISF composition. Bloodstream and lymphatic system may facilitate lymphocyte migration between the blood and peripheral tissues, such as the skin,^29^ resulting in the congruence between the general patterns of PBMC lymphocytes subtypes in dermal ISF. Further examination of the relationship between immune cell subtypes in PBMCs and ISF (Figure 2H) shows that these biological matrices have significantly correlated profiles of CD3+ (in lymphocytes) and CD8+ (in CD3+) through the study time course. At v2, the fractions of IL-17+ (in CD4+) in ISF L tended to reflect those observed in PBMCs, emphasizing the role of this systemic cytokine in AD lesions. For IL-31+ (in CD4+), the opposite was observed, indicating that IL-31 producing cells are enriched in lesions and may not be directly assessed in PBMCs. At the end of the treatment, local CD4+ (in CD3+) tended to increase in circulating immune cells, while they were reduced in the local environment (ISF L).

In our study, the mean percentages of IL-13+, IL-17+, IL-22+, IL-31+, IL-4+, and IL-5+ cells (in CD4+) were decreased in both ISF L and ISF NL at the end of dupilumab treatment, thus suggesting an attenuation of the pro-inflammatory state in the dermis (Figure 2F). The individual trends of CD4+ immune cell subtypes are shown in the Supporting Information (Figure S1)**.**

### 3.2 Profiling of Inflammatory Factors Showed Changes in Local and Systemic Immune Configurations Induced by the Dupilumab Treatment

**Figure 3:**
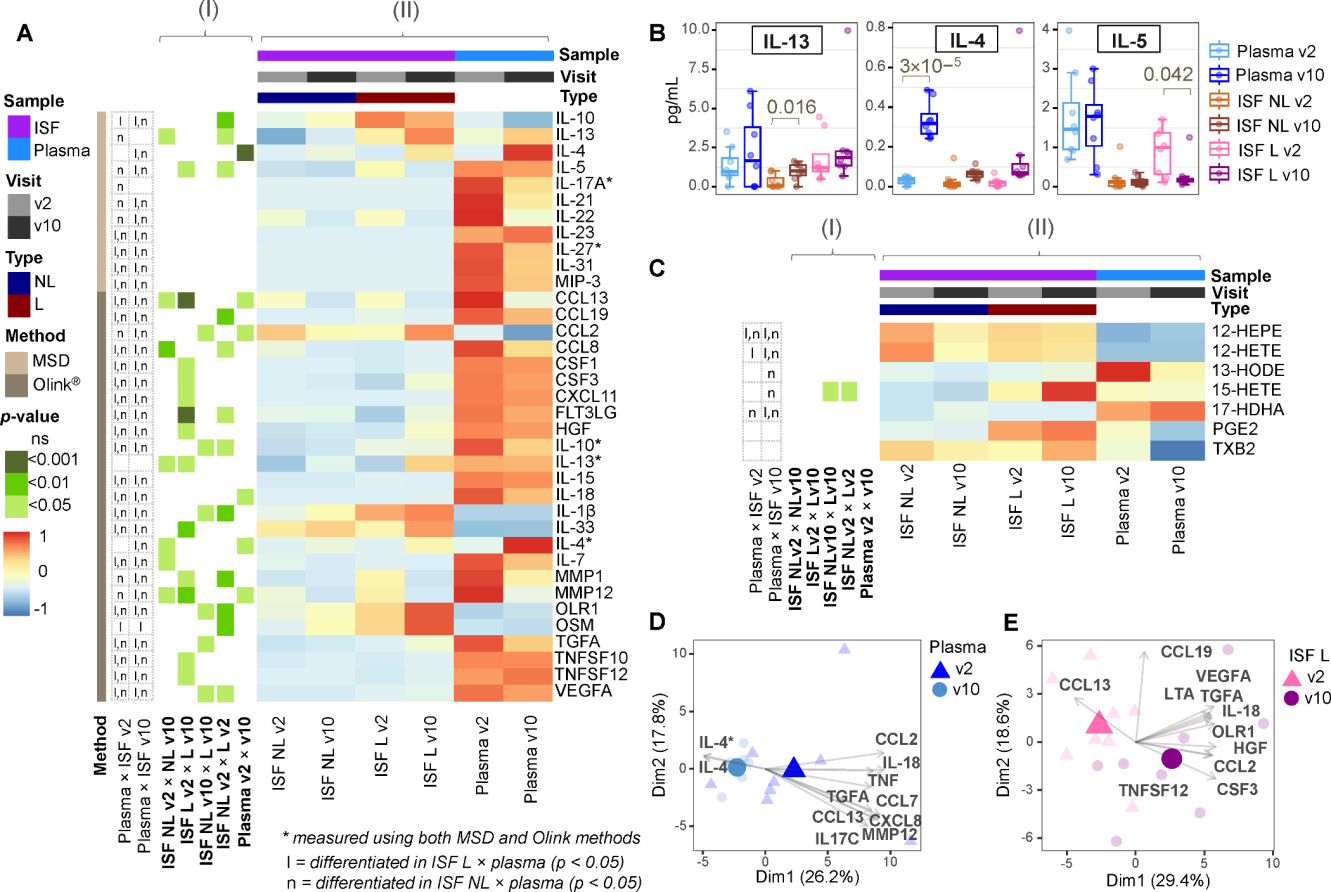
Trends of inflammatory mediators. (A) Heatmaps showing statistically significant differences for the performed comparisons (panel I) and the scaled concentrations of inflammatory factors (panel II) – in the case of the Olink data, only analytes presenting a statistically significant change in at least one of the comparisons are shown. (B) Boxplots depicting the mean concentration values of Th2 interleukins obtained by using the MSD method. (C) Heatmaps showing the level of significance of the performed comparisons (panel I) and the scaled concentrations of eicosanoids in plasma and ISF (panel II). PCA biplots showing the selected most contributing variables characterizing v2/v10 and NL/L in (D) plasma and (E) ISF L, before (v2) and at the end of the treatment (v10). In the PCA biplots, the orientation of an arrow indicates metabolite’s association with the sample group plotted toward the arrow direction (and therefore indicates increased response of the metabolite in this group).

We observed higher mean concentrations of cytokines/chemokines in plasma than in ISF at v2 and at v10, except for IL-1β, IL-33, CCL2, and OLR1 and OSM (Figure 3A), suggesting that these are factors operating locally in the dermis. Both IL-1β and IL-33 are interrelated IL-1 family members. It has been shown that IL-33 is overexpressed in AD keratinocytes.^30^ IL-33 induces IL-31, which in turn stimulates scratching that leads to a further IL-33 release from keratinocytes.^30^ IL-1β is an epidermal cytokine, which locally actuates in skin sensitization and displays upregulated mRNA expression in the case of AD, and which might mediate IL-33 expansion.^31,32^ OSM and CCL2 are expressed by keratinocytes and work on the recruitment of immune cells to the site of the inflammation.^33,34^ OLR1 can also originate in keratinocytes and skin sebocytes, connecting skin disease with dysregulated lipid metabolism.

Dupilumab treatment notably increased IL-4 concentrations in plasma (*p* = 3 × 10^–5^); this was also observed as a trend in ISF samples (Figure 3B). Dupilumab blocks the IL-4Rα receptor, leading to increased concentrations of unbound circulating IL-4 during and after the treatment.^9^ In our study, IL-13 concentrations also indicated an increasing trend at the end of the treatment (v10) versus v2 in all studied samples, with a statistical significance reached for ISF NL (*p* = 0.016) (Figure 3B). In agreement with results from the present study, increased expression of post-treatment IL-13 has been connected with an optimal response to dupilumab, while lower IL-13 and higher levels of type 1 and type 3 cytokines have been observed among nonresponders.^35^ Based on the fact that dupilumab blocks IL-13 signaling, the IL-13 increase at v10 compared to v2 would have been expected to be even more prominent. However, IL-13 can alternatively bind to the IL-13Rα2 receptor, for which associated signaling function is still under investigation.^36^ In the context of the treatment, elevation of IL-4 and IL-13 levels is a phenomenon that has been reported by other trials involving dupilumab.^9,37^ AD lesions are primarily Th2-driven and characterized by increased levels of the corresponding cytokines.^38^ We found significantly reduced concentrations of IL-5 in ISF L at v10 versus v2 (*p* = 0.042, Figure 3B), indicating the cease of IL-5 overproduction at AD lesional sites, which might be linked to less eosinophil activation.

At both time points, the levels of the eicosanoids 12-HEPE, 12-HETE, TXB2, 15-HETE, and PGE2 were higher in ISF L than in plasma samples (Figure 3C). From these eicosanoids, both 15-HETE and PGE2 were particularly elevated in ISF L compared with ISF NL. PGE2 has been reported to be elevated in both lesional and perilesional AD skin.^39^ However, PGE2 signaling has also been demonstrated to negatively regulate AD in murine model by inhibiting the expression of TSLP – one of the cytokines which is generated in keratinocytes and which is believed to induce AD.^39,40^ 15-HETE was more abundant in ISF L than ISF NL, at both v2 and v10 (*p* = 0.011 and *p* = 0.039, respectively, Figure 3C), evidencing its active role in AD lesional sites. Indeed, a previous study has shown that AD lesions exhibit augmented levels of 15-HETE in comparison to nonlesional skin.^39^ Interestingly, 15-HETE mean concentrations in ISF L tended to be higher at v10 in our study. 15-HETE has shown to present inhibitory effects on T-cell proliferation and to suppress the synthesis of leukotriene B4 by leukocytes.^41^

In conclusion, increasing concentrations of 15-HETE and PGE2 from v2 to v10 may indicate an ongoing anti-inflammatory action through the suppression of skin-infiltrating immune cells. On the other hand, at v10, concentrations of 12-HETE and 12-HEPE were decreased in both ISF NL and ISF L. A study exploring filaggrin-mutated AD in human epidermal equivalents has registered augmented levels of these eicosanoids, evidencing enhancement of 12- lipoxygenase (LOX) metabolism in AD; furthermore, 12-HETE and 12-HEPE have lowered the expression of keratinocytes differentiation markers, thus possibly contributing to epidermal dysfunction in AD.^41^ Considering our results, such enhancement of the 12-LOX pathway was potentially reversed by dupilumab treatment. Trends for TXB2 were concordant in both plasma and ISF NL, denoting decreasing concentrations of TXB2 from v2 to v10. A previous study has shown that TXB2 levels were increased in AD serum in comparison to controls, pointing to an augmented activity of the cyclooxygenase (COX) pathway in AD.^42^ Conversely, TXB2 concentrations were increased at v10 versus v2 in ISF L in our study. This diverging trend noticed in lesional samples might be associated with TXB2′s specific role as one of the major itch mediators;^43^ Additionally, TXB2 derives from prostaglandin H2 (PGH2), like PGE2,^43^ suggesting that PGH2 transformation may be a mechanism augmented in ISF L at the end of the treatment.

We investigated further the pattern of inflammatory mediators characterizing the study variables (v2/v10 and NL/L) from a multivariate perspective by using PCA. While plasma at v10 was characterized by an increased IL-4 concentration, the main circulating cytokines at v2 were IL-18 and IL-17C (Figure 3D). Both IL-18 and IL-17C act as enhancers of skin inflammation. Typical AD inflammation seems to be initiated by a broad release of IL-18, which stimulates T cells and mast cells to further release Th2 cytokines and histamine.^44^ In parallel, IL-17C may amplify ongoing epithelial inflammation and immune cell influx in Th2-dominated skin.^45^

At the end of dupilumab treatment (v10), ISF L displayed increased concentrations of factors regulating cell survival, proliferation, migration, and differentiation (Figure 3E). Elevated factors regulating angiogenesis and apoptosis were VEGFA and TNFSF12 (also known as TNF-related weak inducer of apoptosis, TWEAK). TWEAK-induced proliferation of keratinocytes occurs via a pathway mediated by TNF-receptor; moreover, TWEAK appears to potentiate the mitogenic activity of VEGFA.^46,47^ Other factors upregulated in ISF L at v10 versus v2 have been previously associated with lesion repair. HGF has effect upon endothelial and epithelial cells, playing a role in myogenesis and promoting wound healing via β1-integrin/ILK pathway.^48^ CSF3 demonstrated to induce wound repair by promoting keratinocyte proliferation and migration of epithelial cells.^49^ CCL2 appears to be associated with macrophage infiltration during the early inflammatory stages of healthy skin repair, neovascularization, and collagen accumulation.^50^ Interestingly, the concentrations of IL-1 family cytokines IL-1β, IL-18, and IL-33 were increased in ISF L at v10 versus v2, indicating that IL-4Rα inhibition might have a common effect on this group of mediators, which are connected through their receptor structures and signaling transduction pathways.^30^

The present analysis indicated that the number of immune cells (measured using flow cytometry) may not consistently correlate with the cytokine levels in the samples. Functional redundancy, environmental factors, and the type of activation are aspects that may influence cytokine production.^51,52^ Specifically in atopic dermatitis, skin barrier damage and the presence of pathogens may lead to the production of further cytokines derived from keratinocytes.^53^

Between both panels from the Meso Scale Diagnostics (MSD) and the Olink method, five target analytes were the same, and among these, IL-27 concentrations in all ISF samples were below lower limit of quantification for the Olink method. Correlation analysis was used to observe correspondence between analytes measured by both methods. Pearson coefficients (*r*) for IL-10, IL-13, IL-17A, and IL-4 were 0.85, 0.71, 0.87, and 0.99 in ISF L, and 0.92, 0.58, and 0.56 for IL-10, IL-13, and IL-4 in ISF NL, respectively (all *p* < 0.05, except for IL-17A, *p* > 0.05). In plasma, IL-10, IL-4, and IL-27 displayed *r* = 0.93, 0.99, and 0.57, respectively, while IL-13 and IL-17A measurements did not present significant correlation between the MSD and Olink methods (*p* > 0.05). In this way, although it is not possible to compare absolute values provided by the MSD and Olink methods, the trends presented by these analytes are deemed concordant for both methods.

### 3.3 Correlation of Inflammatory Factors and Metabolome Revealed Immune-Metabolic Signatures Relevant for AD Status

**Figure 4:**
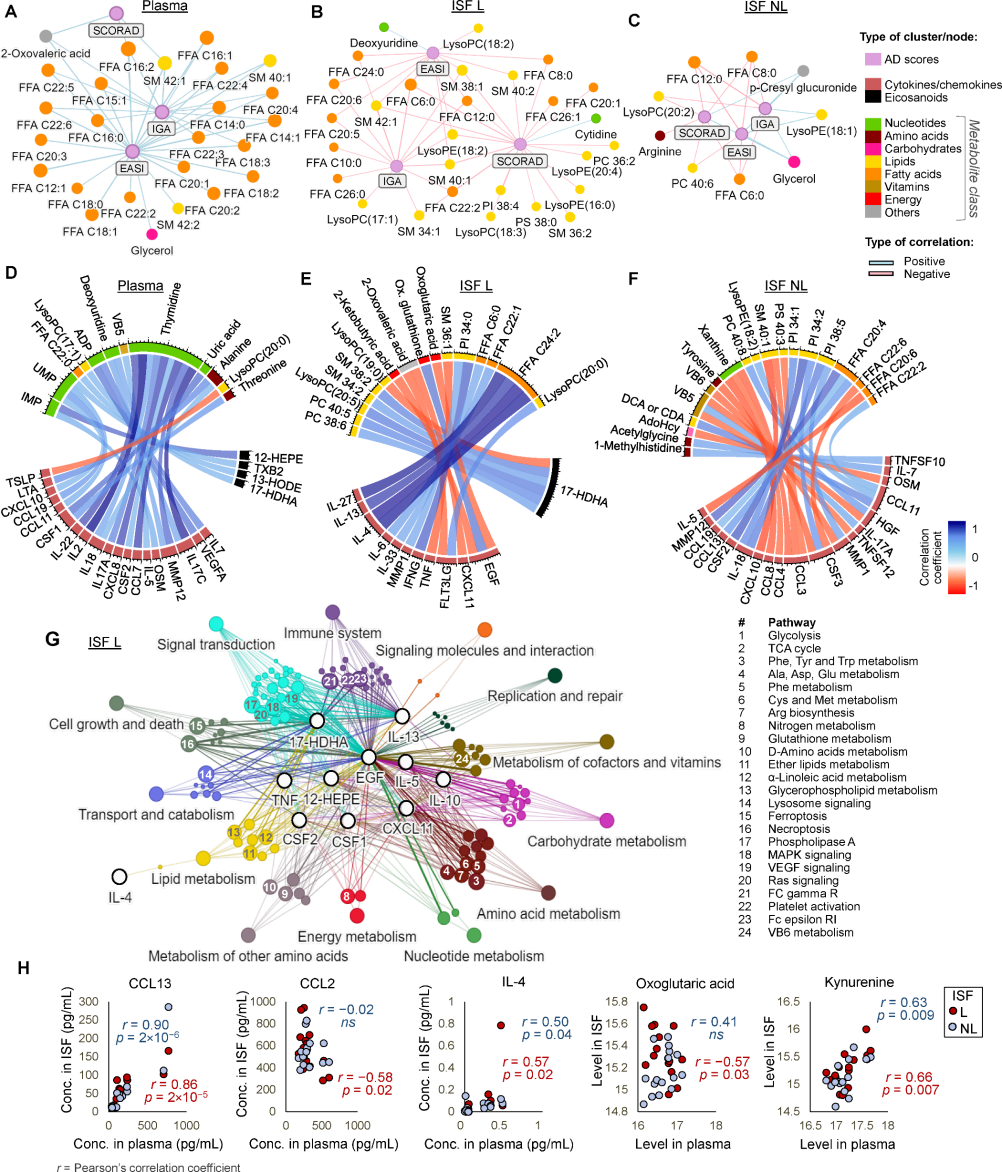
Immuno-metabolic patterns. Networks showing significant correlations (|*r*| > 0.5, *p* < 0.05) between AD scores and experimental data, for (A) plasma, (B) ISF L, and (C) ISF NL. Chord diagrams showing significant correlations (|*r*| > 0.5, *p* < 0.001) between inflammatory factors and metabolites, for (D) plasma, (E) ISF L, and (F) ISF NL. (G) Network showing relationships between inflammatory mediators and pathways in ISF L, according to metabolite enrichment analysis. (H) Trends of cytokines/metabolites in plasma versus ISF at both v2 and v10. FFA = free fatty acid, SM = sphingomyelin, PC = phosphatidylcholine, PI = phosphatidylinositol, VB5 = pantothenic acid, VB6 = pyridoxine, AdoHcy = adenosylhomocysteine, DCA = deoxycholic acid, and CDA = chenodeoxycholic acid.

Next, correlations between the profiles of inflammatory factors, metabolites, and AD scores were investigated in plasma, ISF L, and ISF NL. Specifically in plasma, the levels of several free fatty acids (FFAs), sphingomyelin species, and glycerol were positively correlated with AD scores (Figure 4A). In contrast, long-chain FFAs and several lipids in ISF L were increased, while AD scores decreased, resulting in overall negative correlations (Figure 4B). This suggests that the lowering of AD scores (and therefore improvement of skin condition) is greatly correlated with an increased production of lipids at the lesional site. In ISF NL (Figure 4C), fewer metabolites were associated with AD scores, but most of them were again lipids and shorter-chain fatty acids. As in plasma, glycerol in ISF NL exhibited a positive correlation with AD scores, meaning that this metabolite was decreased in this sample with decreasing AD scores, possibly denoting an increased formation of triacylglycerols (TAGs), components that are known to be linked to the restoration of skin barrier.

In plasma (Figure 4D), a range of inflammatory mediators were positively correlated with the levels of purine nucleotides (ADP, UMP and IMP). Concordantly, it is well-known that inflammatory conditions lead to the extracellular release of nucleotides, required for purinergic signaling.^54^ Still concerning plasma samples, a relevant negative correlation was observed between TSLP and lysophosphatidylcholine (LysoPC) (20:0), suggesting a relationship between lipid metabolism and neuroimmune circuits in AD. TSLP induces the production of the transient receptor potential (TRP) cation channel in sensory neurons and together with other Th2 cytokines may coordinate sensory pathways of itch and inflammation in AD, potentially mediating the cycle of itching and scratching that has been observed among AD patients.^55^ Complementarily, lysophosphatidic acid (LPA), the precursor of LysoPC, has been found to induce itching via TRPA1 signaling.^56^ Although LPA species could not be measured in our metabolomics data set, it was possible to relate LPA to a decreased conversion to LysoPC, hence possibly explaining the negative correlation between TSLP and LysoPC. In ISF L (Figure 4E), pro-inflammatory cytokines, including IL-4 and IL-13, were positively correlated with very long-chain unsaturated fatty acids. In contrast, EGF displayed relevant correlations with energy metabolites. Its association with oxidized glutathione suggests that increased EGF is linked to augmented levels of reactive oxygen species (ROS). This agrees with ROS′ known function as mediators of the EGF/EGFR pathway.^57^ 17-HDHA, a precursor of d-series resolvins, was positively correlated with lipid species of phosphatidylcholine and sphingomyelin, and mediators derived from these lipid species play an important role in inflammation. ISF NL (Figure 4F) was characterized by different associations between chemokines and other factors with FFAs and lipids, mainly PI species. Such connections suggest the relevance of the lipid-chemokine-cytokine cascade in skin inflammation. Most relevant correlations between cytokines/chemokines and metabolites in plasma were positive, while in ISF, these were either positive or negative. This aspect highlights the difference between the dynamics of systemic and local inflammatory signaling, particularly because ISF represents the dermis, where extensive pathophysiological changes were associated with by the dupilumab treatment.

PEA was performed for metabolites significantly correlated with the levels of inflammatory mediators at v2 and v10 in ISF L, in order to assess how local immune profiles and metabolism are interrelated in lesional AD. The associations between inflammatory factors and metabolism in ISF L during the dupilumab treatment are depicted in the form of networks (Figure 4G). EGF was the factor mobilizing a broader variety of metabolic pathways. Once EGF stimulates cell growth, the influence on various biosynthetic pathways and signaling interactions is expected. Since IL-4 signaling is inhibited during the treatment, pathways typically associated with its function could not be mapped. However, IL-4 was only correlated with lipid metabolism, probably as a result of skin improvement being connected with enhanced lipid synthesis. Conversely, pathways appearing connected with IL-13 are likely associated with the effects of its binding to the alternative receptor (IL-13Rα2).

Ultimately, a correlation analysis for variables in plasma and ISF exemplifies concordances and differences between the assessed biomaterials (Figure 4H). CCL13, a chemoattractant for a series of white blood cells that was increased in v2 versus v10 in all studied biofluids, displayed ISF L and ISF NL levels that were strongly correlated with plasma. For CCL2, a significant negative correlation was observed in lesional samples and plasma, as this cytokine appeared particularly enriched in ISF L. Finally, it can be noticed that IL-4 plasma levels have a moderate but significant association with IL-4 trends in ISF L and ISF NL. The particular enrichment of oxoglutaric acid (α-ketoglutaric acid) in lesional samples led to a significant negative correlation with plasma levels. Finally, kynurenine exhibited concordant levels in all assessed matrices. Both compounds are examples of the crosstalk between metabolism and inflammation signaling.^58,59^

### 3.4 Dupilumab Treatment Altered Metabolic Signatures Associated with the AD Phenotype in ISF

#### Metabolome Retrieved from ISF Was More Informative than Plasma and Metabolic Changes Were More Pronounced in Lesional Skin

**Figure 5:**
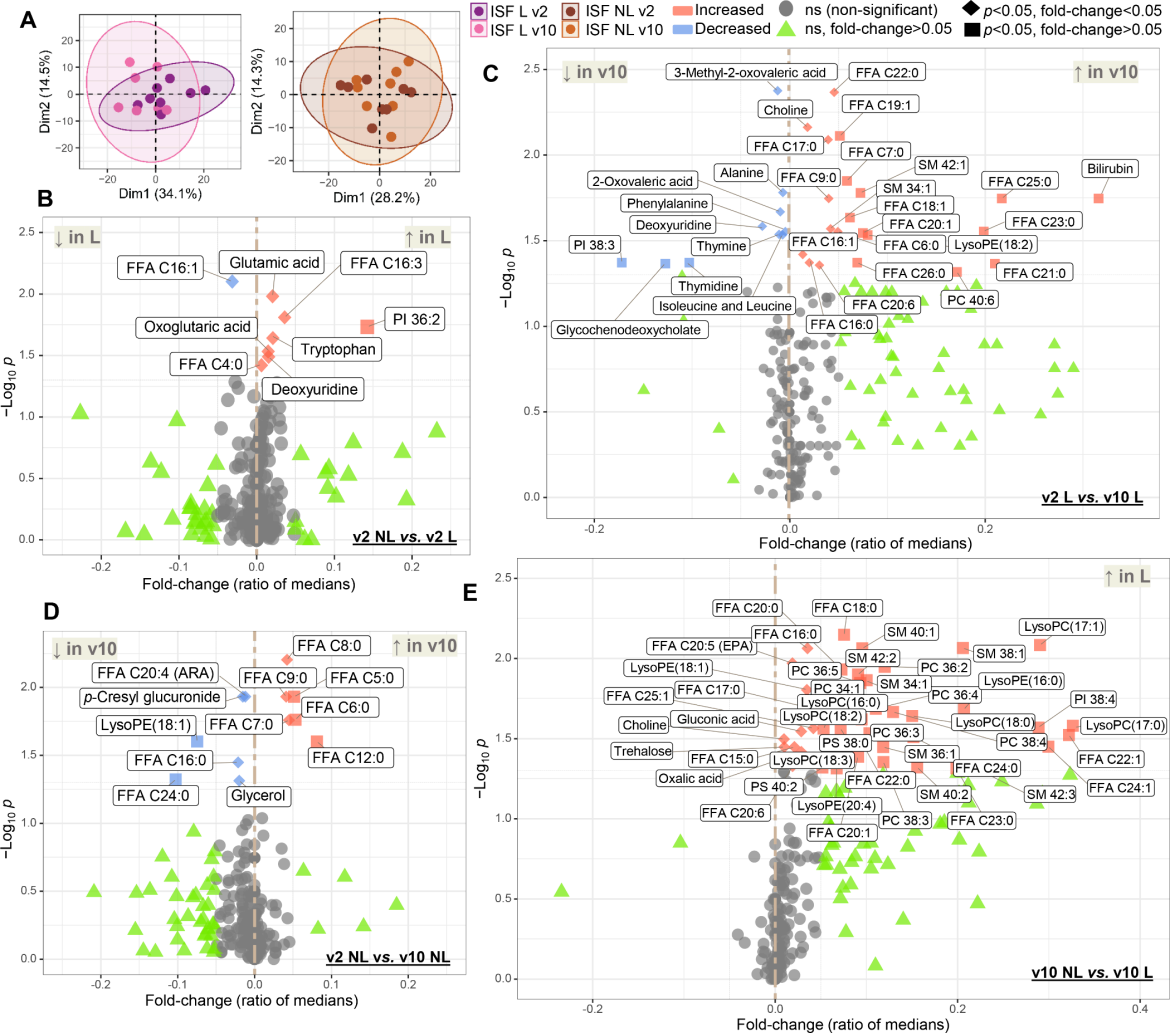
ISF metabolomics. (A) PCA score plots for ISF metabolome at v2 and v10; volcano plots representing results of univariate analysis when comparing (B) NL versus L sites at v2, (C) L sites at v2 and v10, (D) NL sites at v2 and v10, as well as (E) NL versus L sites at v10. FFA = free fatty acid, SM = sphingomyelin, PC = phosphatidylcholine, PI = phosphatidylinositol, PE = phosphatidylethanolamine, and PS = phosphatidylserine.

Metabolite patterns of ISF L were more affected by the dupilumab treatment than those of ISF NL, for which samples at v2 and v10 displayed greater overlap (Figure 5A). A statistical comparison of plasma samples obtained at v2 and v10 (Figure S2) did not present relevant statistically significant changes. However, application of a lowered significance criterion (*p* < 0.1) suggested that the treatment led to decreased levels of several long-chain FFAs (LCFAs, mainly unsaturated ones) and carnitine. Overall, these results indicate a systemic change in lipid metabolism and beta-oxidation at the end of dupilumab treatment.

The availability of ISF samples allowed direct assessment of differences between lesional and nonlesional skin and thus enabled a detailed discussion based only on statistically significant metabolic changes.

#### Metabolic Patterns Characteristic for AD Lesions Included Lipid Signaling and Amino Acid Metabolism

Volcano plots (Figure 5B–E) provide a combined representation of the magnitude and significance of metabolite changes in ISF samples. At v2, the decreased levels of palmitoleic acid in ISF L in relation to ISF NL (Figure 5B) can be due to its recruitment as a lipokine but also as a consequence of abnormalities in fatty acid/lipid pathways in AD. It has recently been shown that the lipokine phosphatidylinositol (PI) (36:2–18:1/18:1) regulates cellular stress by suppressing p38 mitogen-activated protein kinase activation.^60^ Our data showed that at v2, PI 36:2 levels were significantly pronounced in lesional skin (Figure 5B), which may denote resistance against stress signaling derived from on-site inflammation. Glutamate is among the main constituents of filaggrin, and it is a precursor of the natural moisturizing factor.^61,62^ Increased glutamic acid observed in ISF L in comparison to ISF NL at v2 can be linked to enhanced filaggrin breakdown that tends to occur under dry conditions, typical of AD skin. In addition, glutamate has also been referred to as a neuromediator involved in pruritus during AD.^63^ In our study, increased tryptophan levels were also observed in ISF L in comparison to ISF NL at v2. In a superficial examination, decreased tryptophan levels following dupilumab treatment could be expected in lesional samples, as it has been demonstrated that inflammatory mediators induce indoleamine 2,3-dioxygenase (IDO) activity, which is an enzyme responsible for tryptophan oxidation in the kynurenine pathway.^64,65^ However, it has been shown that tryptophan degradation rate may also be affected by the downstream metabolite quinolinic acid, which induces nitric oxide synthase (NOS).^14^ In its turn, nitric oxide can suppress IDO activity, resulting in elevated concentrations of tryptophan.^66^ Additionally, the preferential upregulation of kynureninase (an enzyme subsequent to IDO in the kynurenine pathway) in AD lesions has been reported.^58,67^ Previous research has demonstrated that IL-4 activates SUMO Specific Peptidase 1 - Sirtuin 3 (SENP1-Sirt3) signaling, which enhances glutamate dehydrogenase (GLUD1) activity in glutaminolysis, triggering oxoglutarate accumulation and thus leading to M2 macrophage polarization.^59^ Therefore, the positive modulation of glutamate and oxoglutarate in ISF L at v2 is aligned with metabolic shifts connected with immunological/inflammatory processes previously described.

#### ISF from Lesions Indicated Downregulation of Inflammatory Pathways at the End of the Treatment

In AD, disturbance of the epidermal barrier function has been shown to be accompanied by keratinocyte apoptosis.^68^ In ISF L at v10 (Figure 5C), we observed significant decrease in four possible products of nucleic acids breakdown (thymine, thymidine, deoxyuridine, and alanine), hence indicating decreased apoptosis. 3-Methyl-2-oxovalerate is a branched-chain amino acid possibly derived from isoleucine. Branched-chain amino acids promote ROS production and trigger NF-κB activation.^69^ Additionally, urinary 3-methyl-2-oxovalerate has been found to be positively associated with the occurrence of food sensitization and serum IgE.^69,70^ Both 3-methyl-oxovalerate and isoleucine levels were decreased in ISF L at v10 in our study, indicating the retraction of the inflammatory processes. Bilirubin levels at v10 in ISF L displayed a 3-fold increase in relation to v2. Bilirubin is known for serving as an endogenous antioxidant and therefore performing cytoprotective functions.^71^ Concordantly, bilirubin appears to be associated with the IL-10 anti-inflammatory pathway.^72^ IL-10 induces heme oxidation, which releases bilirubin but also carbon monoxide which may inhibit inflammatory cytokines.^72,73^ Indeed, in our data, bilirubin levels displayed a significant negative correlation with IL-10 levels in ISF L (*r* = −0.55, *p* = 0.03). A previous study has shown that biopyrrin levels were augmented in AD cells while bilirubin levels were decreased, thus evidencing bilirubin oxidation in AD lesions.^74^

Glycochenodeoxycholic acid levels were decreased at ISF L in v10 in comparison to v2 (Figure 5C), indicating further ramifications of AD in the biliary metabolism. Other studies have shown abnormal bile acids patterns in the plasma of psoriasis patients.^75,76^ Primary bile acids may modulate immune response inducing an anti-inflammatory phenotype characterized by an increased IL-10/IL-12 ratio through PKA activation.^77^ Complementarily, administration of bile acids has attenuated psoriasiform dermatitis in a mouse model due to direct inhibition of IL-17A and CCL20.^78^ In this manner, decreasing glycochenodeoxycholate levels may reflect the depletion of an ongoing systemic anti-inflammatory mechanism before the treatment.

#### Impairments in AD Lipid Synthesis Were Possibly Overcome at the End of the Treatment

Disturbances in lipid profiles appear to be closely related to the impairment of epidermal barrier functions observed in AD.^79^ In ISF NL, glycerol concentrations were lowered at v10 (Figure 5D) consistent with a generalized decrease in glyceride breakdown as well as with enhanced biosynthesis of glycerophospholipids. Complementarily, in our data, glycerol was negatively correlated with free IL-4 and IL-13 (*r* = −0.50 and −0.58, and *p* = 0.049 and 0.019, respectively). Overall, the treatment was associated with an upregulation in lipid biosynthesis which was more accentuated in ISF L than in ISF NL (Figure 5C,E). In our data, the sphingomyelins 34:1, 42:1, and phosphatidylcholine 40:6 were elevated in ISF L at the end of treatment, while sphingomyelins 42:1, 38:1, 40:1, 42:2, 42:3, and phosphatidylcholines 34:1, 36:2, 36:3, 36:4, 36:5, and 38:4 at v10 were particularly increased in ISF L compared to ISF NL. In AD, sphingomyelin and phosphatidylcholine profiles may be affected due to dysfunctions observed in competent enzymes.^80−82^ Similar findings were reported by a previous study, in which AD patients, which responded to anti-IgE antibody therapy, displayed increased serum levels of glycerophospholipids, in particular phosphatidylcholines.^83^ This upregulation in lipid synthesis at the end of the treatment may also be associated with an increased demand for structural molecules needed for the restructuring of *stratum corneum* lipid matrix and the growth of new keratinocytes.^84^

#### Biosynthesis of Long/Very Long-Chain FAs (LCFA/VLCFA) Was Enhanced at the End of the Treatment

The levels of species containing longer chain fatty acids were significantly increased in ISF L at the end of dupilumab treatment (sphingomyelins 34:1, 42:1, and phosphatidylcholine 40:6) (Figure 5C). The same was observed for longer chain FFAs C18:1, 20:1, 20:6, 22:0, and 26:0. Fatty acid synthesis in AD appears to be characterized by impaired production of LCFAs and very long-chain fatty acids (VLCFAs).^85^ IL-13 and IL-4 signaling has shown to reduce the expression of elongation of VLCFA protein (ELOVL) 3 and ELOVL6 in keratinocytes *in vitro*, leading to a decreased fraction of LCFAs in ceramides and sphingomyelins.^85^ Additionally, ELOVL1 expression has appeared to be reduced in AD lesional skin.^86,87^ In our study, IL-13 levels were correlated with the levels of several LCFAs, such as the FFA C16:0, 18:0, 22:0 (*r* = 0.52–0.54, *p* = 0.035–0.045) in ISF L. Furthermore, both IL-4 and IL-13 were associated with other VLCFAs such as FFA C22:1 and 24:2 (*r* = 0.57–0.98, *p* = 0.026–6 × 10^–10^). Therefore, ELOVL inhibition appears to cease at the end of the treatment, allowing increased production of LCFA/VLCFAs in ISF L, in comparison to v2 and in comparison to ISF NL at v10 (Figures 5C and 4E).

#### Dupilumab Treatment Upregulated the Biosynthesis of Unsaturated FAs and Lowered Beta-oxidation, Particularly in Lesional Skin

Parallel with the decrease in the levels of FFA C16:1 in ISF L versus ISF NL at v2 (Figure 5B), an increase in the levels of unsaturated FFAs (e.g., FFAs C16:1, 18:1, 20:1, 20:6) (Figure 5C) was observed at the end of the treatment (v10). Also at v10, other unsaturated FFAs (e.g., C20:5, 20:6; 22:1; 24:1) were augmented in ISF L in comparison to ISF NL (Figure 5E). This indicates the upregulated biosynthesis and recruitment of such species at the lesional sites, where they may play the role as signal transducers and are also required to prevent epidermal water loss.^88^ Previous studies have reported an increased activity of the epidermal form of fatty acid-binding protein (FABP) in dermatitis and other skin disorders.^89,90^ Since FABP preferentially binds to saturated fatty acids,^91^ this could contribute to a lower traffic of unsaturated FFAs at v2 when compared to v10. Other proteins supporting beta-oxidation are known to be overexpressed in skin diseases, i.e., carnitine palmitoyltransferase-1 (CPT-1) and peroxisomal fatty acyl-CoA oxidase (ACOX1).^92,93^ This is congruent with the carbon chain shortening lipid profile observed in the present study, indicating that beta-oxidation may be an important source of energy for supplying the intensified and continuous cell growth in lesional skin. A number of saturated fatty acids able to undergo beta-oxidation were elevated at v10 in ISF L when compared to v2 (e.g.: FFA C22:0 and 26:0) and when compared to ISF NL (e.g.: FFA C24:0, 22:0, 20:0 and 18:0), suggesting the regulation of the fatty acid catabolism.

#### AD Lipid Metabolism Associated with Inflammation Was Altered at the End of the Treatment

Considering ISF NL, arachidonic acid (FFA C20:4) was present in significantly lower levels at v10 than at v2 (Figure 5D), indicating that the propagation of the arachidonic acid pathway was suppressed at the end of the treatment. LysoPC species are lipid second messengers, possibly chemoattractants for T cells, which at v10 were increased in ISF L versus ISF NL (Figure 5E). Decreased contents of LysoPCs have been noticed in the case of AD skin, potentially reflecting in a reduced innate immunity.^87^ Therefore, it is possible that the production of LysoPCs was increased at v10 due to the restitution of normal lipid metabolism and also as a mechanism supporting immune response. Greater levels of LysoPC in ISF L may also be due to a higher activity of phospholipase 2 (PLA2) in lesional sites, once this enzyme is induced by factors linked to tissue injury.^94^

### 3.5 ISF Analysis Revealed Changes in Locally Relevant Signaling Pathways and Gene Expression at the End of the Treatment

#### Enrichment of Metabolomics Data Showed That the Treatment Affected Core Metabolic Routes and Signaling Pathways

**Figure 6:**
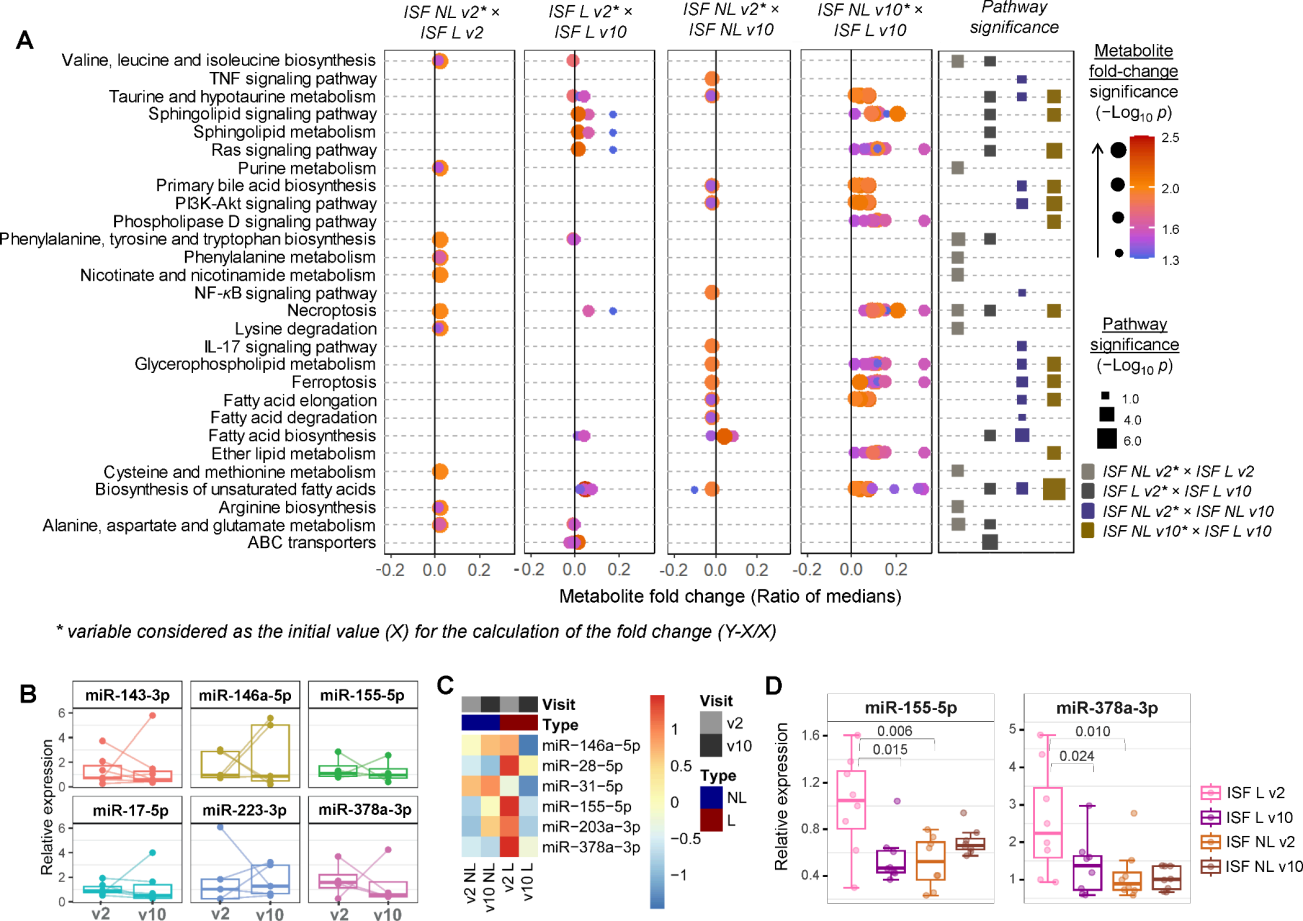
Pathway analysis and miRNA trends. (A) PEA results–the first four plots show the trends (fold changes and their significances) presented by metabolites ascribed to each pathway. Increased metabolites were plotted at the left, and decreased ones were plotted at the right of the *x* = 0 line. Reddish dot colors and greater dot sizes refer to more significant changes in metabolite response (lower *p* values) for each comparison. The last plot expresses the significance of the pathways with greater squares indicating lower *p* values obtained for a given pathway based on hypergeometric testing. (B) Parallel coordinates plot of miRNAs expression in lesional skin biopsies at v2 and v10. (C) Heatmap displaying average expression of miRNAs in ISF L and NL, at v2 and v10. (D) Boxplots showing ISF miRNAs with statistically significant differences in their expressions.

When comparing ISF NL and ISF L at v2, the most relevant affected pathways were the metabolism of purines and amino acids, particularly the aromatic amino acids (which includes the kynurenine pathway). Nicotinate and nicotinamide metabolism (NNM) was another pathway mapped accordingly with PEA, specifically enhanced in ISF L (Figure 6A). In NNM, nicotinamide phosphoribosyltransferase, the rate-limiting enzyme in the salvage of NAD^+^, has been found induced in lesional AD, thus correlating NAD^+^ metabolism with oxidative stress and skin inflammation in AD.^95^ Besides that, in our study, levels of metabolites participating in necroptosis were also increased in ISF L when compared with ISF NL at both v2 and v10. This suggests the intensification of regulated necrotic cell death, specifically at lesional sites. ISF L at v10 compared to v2 showed changes in pathways related to amino acids (amino acid metabolism and ABC transporters), in addition to the positive modulation of sphingolipid metabolism and signaling and biosynthesis of unsaturated FFAs. Increasing metabolite trends were also observed for the Ras signaling pathway, which appears to coordinate keratinocyte and epidermal proliferation, thus demonstrating to be essential for proper skin development.^96^ Moreover, at v10, Ras signaling appeared upregulated in ISF L in relation to ISF NL, suggesting that this pathway may play specific roles in AD lesions.

In the comparison of ISF NL at v2 and v10, relevant pathways were the PI3K-Akt signaling pathway, ferroptosis, and FFA biosynthesis (particularly for unsaturated ones). It has been demonstrated that in AD peripheral T cells, the PI3K/Akt pathway is overactive, inducing T cell proliferation and the secretion of cytokines such as IL-6 and IL-10.^97^ Ferroptosis has shown to regulate normal epidermal differentiation.^98^ Connected with this, imbalances in ferroptosis have been associated with AD, and could prompt disruptions in skin homeostasis.^99^ Hence, based on these results we assume that PI3K-Akt and ferroptosis pathways were probably also enhanced in nonlesional AD skin, but were lessened at the end of treatment. On the other hand, comparing ISF L and ISF NL at v10, these pathways remain upregulated explicitly in lesions. It is also of notice that PEA points to a downregulation of IL-17 and NF-κB pathways at v10 in ISF NL. This is due to the decreasing trend displayed by arachidonic acid in ISF NL, the only metabolite ascribed to the aforementioned pathways. In correspondence with univariate analysis of metabolites, PEA of ISF L at v10 was characterized by increased activities of fatty acid biosynthesis and elongation, as well as metabolism of glycerophospholipids and sphingolipids. The phospholipase D pathway was also enriched according to PEA in our study as a consequence of very active lipid metabolism in ISF L at v10, indicating that many lipid species that were elevated in lesions participate in signaling associated with wound repair. It was previously demonstrated that phospholipase D activation may play a role in membrane repair and can induce the production of phosphatidylglycerol, promoting wound healing.^100^

#### MiRNAs Normally Upregulated in AD Showed Reduced Expression in ISF at the End of the Treatment

In skin biopsies (only L skin site), no clear trends could be observed regarding the differential expression of miRNAs before and at the end of the treatment (Figure 6B). This may be due to the small sample size or variability in cell content of the collected skin biopsy samples. Among miRNAs analyzed in ISF, miR-146a and miR-28–5p in ISF L were decreased at the end of treatment, although the changes were not statistically significant (Figure 6C). MiR-146a levels have been reported to be increased in AD keratinocytes, its upregulation is related to the inhibition of pro-inflammatory factors, as it inhibits multiple genes from the NF-kB pathway.^101^ Regarding miR-28–5p, it has been proposed to target STAT5B, which plays a role in skin inflammation by regulating memory T cells, activating mast cells, and being required for IL-22 production.^102^ Such trends of miRNAs expression indicate that the dupilumab treatment led to the downregulation of a dynamics of gene expression control counteracting the pro-inflammatory phenotype in AD. MiR-203a-3p was also decreased in ISF L at v10 in our study. This is a keratinocyte-specific miRNA and has found to be elevated in AD serum,^103^ thus likely being relevant in AD pathogenesis. MiR-203 has been described as an inducer of keratinocyte differentiation and cell-cycle exit, being a suppressor of p63–a regulator of epidermal differentiation.^104^ In addition, miR-203 has been shown to target suppressor of cytokine signaling (SOCS) 3 and thereby to contribute to the development of psoriasis.^103^ It can be assumed that miR-203a-3p develops roles in AD progression, which were reinforced by the proinflammatory phenotype, or it can contribute as inducer of inflammation similarly to its proposed function in psoriatic skin.

At v2, miR-155–5p and miR-378a-3p were both significantly elevated in ISF L compared to ISF NL (*p* = 0.006 and *p* = 0.010, respectively). These miRNAs were also notably decreased in ISF L at v10 compared to v2 (*p* = 0.015 and *p* = 0.024, respectively) (Figure 6D). A previous study has reported miR-155 overexpression in AD lesions, while its expression in healthy skin has been relatively low.^104^ This miRNA has been shown to inhibit SOCS1 in multiple cell types, c-Maf (a transactivator of IL-4 promoter) and CTLA4 in Th cells, thereby attenuating Th2 response.^105,106^ Correspondingly, Th cells have been determined as the major contributors to miR-155 overexpression in AD.^104^ Under dupilumab treatment, less infiltrating Th cells can be a cause for reduced miR-155 expression in our data. MiR-378a-3p augmented expression has been associated with both AD and psoriasis.^102,107^ Interestingly, miR-378a-3p has been shown to indirectly increases multiple pro-inflammatory cytokines in keratinocytes upon IL-17 treatment.^107^ On the other hand, miR-378a-3p in inflamed colonic mucosa have shown to be inversely correlated with IL-33 mRNA and protein;^108^ accordingly, the same trend was observed in relation to miR-378a-3p expression and IL-33 levels in our results, emphasizing the possible regulatory function of this miRNA in inflammation.

This study presents possible limitations regarding the small sample size and the lack of serial time points for sample collection. On the other hand, a thorough selection of participants was performed to minimize variabilities within the characteristics of the study group and to ensure the successful implementation of dOFM with this purpose. A longitudinal study involving more participants and the evaluation of biological samples at different time points should be considered as a future direction focused on understanding temporal patterns in local metabolism reprogramming. Despite the study’s limitations, changes in the levels of biological entities were supported by significant statistical results from different data analysis strategies. Although preliminary, this study describes an innovative approach, and the findings justify the promotion of large-scale investigations.

## 4 Conclusions

Dermal ISF obtained using dOFM sampling has proven to be suitable for obtaining high-resolution profiles of immune cells, cytokines/chemokines, eicosanoids, miRNAs, and a wide range of metabolites, highlighting the promising potential of dOFM for *in vivo* monitoring in the clinical setting. Dupilumab treatment led to a significant clinical improvement of AD in all study participants. Differences in molecular profiles before and after treatment (v2 versus v10) underline mechanisms involved in the amelioration of AD, demonstrating how the therapy impacted disease pathology at a molecular level. Among these, the following shall be highlighted: (i) effective IL-4Rα blockade, denoted by increased levels of free IL-4 and IL-13; (ii) acquisition of a less inflammatory immunophenotype; (iii) enabling of skin repair and re-epithelialization, evidenced by increased levels of factors promoting keratinocyte proliferation and angiogenesis; (iv) return to metabolic homeostasis, as the obtained local metabolome indicated reduced apoptosis, oxidative stress, and beta-oxidation; (v) improved skin barrier function, indicated by locally elevated levels of LCFAs, VLCFAs, unsaturated FFAs, and lipids, which also correlated with lower AD scores; vi) reprogramming of the local gene expression, indicated by the reduced expression of AD-associated miRNAs. In summary, results from the present study provided novel insights by linking local immune and metabolic alterations to AD pathogenesis and the treatment response.

## Data Availability

The mass spectrometry raw data are available in the Zenodo repository (DOI: 10.5281/zenodo.11261051).

## Supporting Information Available

Table S1: study inclusion and exclusion criteria; Table S2: flow cytometry: gating strategy for deep immunophenotyping (surface markers); Table S3: flow cytometry: gating strategy for cytokine producing cells (intracellular markers); Figure S1: changes in the populations of CD4+-derived interleukins at baseline (v2) and at the end of treatment (v10) in ISF L and ISF NL; Figure S2: metabolite changes in plasma samples at v10 in relation to baseline (v2) (PDF)
Table S4: results of normality tests (XLSX)
Table S5: parametric tests results for miRNAs and inflammatory mediators (XLSX)
Table S6: nonparametric tests results for miRNAs and inflammatory mediators (XLSX)
