# Supplementary material for: Characterization of Inflammatory Mediators and Metabolome in Interstitial Fluid Collected with Dermal Open Flow Microperfusion before and at the End of Dupilumab Treatment in Atopic Dermatitis
Source: J Proteome Res. 2024 Jul 10;23(8):3496–514. doi: 10.1021/acs.jproteome.4c00153 (PMC11304394; doi:10.1021/acs.jproteome.4c00153)
Supplement: Supplementary file 1 — pr4c00153_si_001.pdf [file pr4c00153_si_001.pdf]

Supporting information

Characterization of inflammatory mediators and metabolome in interstitial fluid collected with dermal open-flow microperfusion (dOFM) before and at the end of dupilumab treatment in atopic dermatitis

Fernanda Monedeiro[†1], Barbara Ehall[†2,3], Katrin Tiffner[1], Anita Eberl[1], Eva Svehlikova[2], Barbara Prietl[2,4], Verena Pfeifer[2,4], Julia Senekowitsch[2], Anu Remm[5], Ana Rebane[5], Christoph Magnes[1], Thomas Pieber[1,2,4], Frank Sinner[1,2], Thomas Birngruber[1*]

1 Joanneum Research Forschungsgesellschaft mbH, HEALTH – Institute for Biomedical Research and Technologies, Graz, Austria

2 Division of Endocrinology and Diabetology, Medical University of Graz, Graz, Austria

3 BioTechMed, Graz, Austria

4 Center for Biomarker Research in Medicine (CBmed) GmbH, Graz, Austria

5 Institute of Biomedicine and Translational Medicine, University of Tartu, Estonia

† These authors contributed equally to this work

* Corresponding author

Email: Thomas.Birngruber@joanneum.at

Table of Contents

Table S1 – Study inclusion and exclusion criteria....S2

Table S2 – Flow cytometry: Gating strategy for deep immunophenotyping (surface markers) S4

Table S3 – Flow cytometry: Gating strategy for cytokine producing cells (intracellular markers) S5

Figure S1 – Changes in the populations of CD4+-derived interleukins at baseline (v2) and at the end of treatment (v10) in ISF L and ISF NL. S6

Figure S2 – Metabolite changes in plasma samples at v10 in relation to baseline (v2)....S6

Table S4 – Results of normality tests

Table S5 – Parametric tests results for miRNAs and inflammatory mediators

Table S6 – Nonparametric tests results for miRNAs and inflammatory mediators

Table S1 – Study inclusion and exclusion criteria

**Inclusion Criteria**	
1	Male and female atopic dermatitis patients at an age of 18 to 65 years (both inclusive) at the time of signing the informed consent.
2	Chronic atopic dermatitis (AD) diagnosed for at least six months before enrollment (according to American Academy of Dermatology Consensus Criteria and to the UK Working Party's diagnostic criteria for atopic dermatitis)
3	Moderate-to-severe atopic AD classified as<br>• EASI (Eczema Area and Severity Index) score >16 and<br>• IGA (Investigator's global Assessment) score ≥ 3 and<br>• not adequately controlled by topical medication.
4	At least one suitable AD lesion that is well accessible for the dOFM investigation.<br>For insertion of one dOFM probe a lesion of at least 35 mm length and 10 mm width is required. dOFM placement can be as follows: All dOFM probes can be placed in one lesion, each dOFM probe can be inserted in an individual lesion, or two probes can be placed in one lesion and two in another.
5	The participant is able to understand the study requirements, procedures, risks and benefits as outlined in the participant information sheet and informed consent form, is willing and able to comply with the specified requirements and procedures, and has signed the informed consent form before any trial-related activities. Trial-related activities are all procedures that are carried out as part of the trial, including activities to determine the participant´s suitability for the trial.
6	If the participant is female of child-bearing potential, she must have a negative serum beta human chorionic gonadotropin (β-HCG) pregnancy test performed within 21 days prior to v2 and use an adequate and acceptable method of birth-control during the study.<br>A female participant is considered of non-childbearing potential if one of the following is reported and documented on the medical history:<br>postmenopausal with spontaneous amenorrhea for at least one (1) year, or<br>surgical sterilization
7	The participant is available for the entire study duration
**Exclusion Criteria**	
1	Pregnant and breast feeding women and women unwilling to use reliable contraception during the study, if of child-bearing potential and sexually active.
2	Significant allergies to humanized monoclonal antibodies, known hypersensitivity to dupilumab or any of its excipients.
3	Clinically significant multiple or severe drug allergies, or severe drug-induced hypersensitivity reactions (including, but not limited to, erythema multiforme major, linear immunoglobulin A dermatosis, toxic epidermal necrolysis, and exfoliative dermatitis).<br>Active dermatoses in need of systemic treatment.
4	History of an ongoing, chronic or recurrent infectious disease, or known tuberculosis infection (both active and previous) as orally stated by the participant;<br>History of Hepatitis B or C or HIV or positive screening serology test for Hepatitis B, C (acute infection) or HIV.

5	Alanine transaminase (ALT) > 2 x upper limit of normal (ULN).
6	Bilirubin > 1.5 x ULN (isolated bilirubin > 1.5 x ULN is acceptable if bilirubin is fractionated and direct bilirubin < 35%).
7	Current unstable liver or biliary disease per investigator assessment defined by the presence of ascites, encephalopathy, coagulopathy, hypoalbuminaemia, oesophageal or gastric varices, persistent jaundice, or cirrhosis. NOTE: Stable chronic liver disease (including Gilbert's syndrome, asymptomatic gallstones) is acceptable if the participant otherwise meets the entry criteria.
8	Asthma treated with other than inhalator medication (i.e. per oral or intramuscular anti-asthmatic medication is not allowed)
9	Known helminth infection, diagnosed active endoparasitic infections; suspected or high risk of endoparasitic infection, unless clinical and (if necessary) laboratory assessments have ruled out active infection.
10	Plans for administration of live vaccines during the study period or 4 weeks after last visit or within 12 weeks prior to dosing.
11	Treatment with biologic agents (such as monoclonal antibodies including marketed drugs) within 16 weeks or 5 half-lives (whichever is longer) or cell-depleting agents within 6 months before baseline, or until lymphocyte count returns to normal (whichever is longer) prior to dosing.
12	Treatment with dupilumab within 16 weeks prior to dosing.
13	Immunosuppressive/immunomodulating drugs (systemic corticosteroids, cyclosporine, mycophenolate-mofetil, IFN-γ, Janus kinase inhibitors, azathioprine, methotrexate) or phototherapy for AD within 4 weeks before v2, or any condition that in the opinion of the investigator, is likely to require such treatments during the first 4 weeks of study treatment.
14	Treatment with topical corticosteroids or topical calcineurin inhibitors within 1 week before screening.
15	Excessive sun exposure, e.g regular use (more than 2 times per week) of a tanning booth/parlour within 4 weeks prior to first treatment and during the whole study treatment period.
16	Treatment with any other medications for AD that could interfere with efficacy outcomes or affect the evaluation for AD severity.
17	Uncontrolled systemic disease and ongoing infections (active chronic or acute infection requiring treatment with systemic antibiotics, antivirals, antiparasitics, antiprotozoals, or antifungals within two weeks prior to v2 or superficial skin infections within one week prior to the v2)
18	Known or suspected history of immunosuppression, including history of invasive opportunistic infections (e.g., tuberculosis, histoplasmosis, listeriosis, coccidiomycosis, pneumocystis, aspergillosis) despite infection resolution; or unusually frequent, recurrent, or prolonged infections, per investigator judgement.
19	History of malignancy within five years before the screening visit, except completely treated in situ carcinoma of the cervix, or completely treated and resolved non-metastatic squamous or basal cell carcinoma of the skin.
20	Smoker who is not willing to refrain from smoking during the in-house dOFM sampling visits.
21	History of drug and/or alcohol abuse within one year of v1.
22	Planned or anticipated major surgical procedure during the patient's participation in this study.
23	Positive result of urine drug screen.
24	Positive alcohol breath test.
25	Presence of skin comorbidities that may interfere with study assessments.
26	Any reason which, in the opinion of the investigator, would prevent the participant from safely participating in the study.
27	Any abnormalities found at physical examination or vital signs, unless deemed not clinically significant by the investigator.
28	Clinically significant abnormal laboratory evaluation results, as deemed by the investigator.

29	Clinically significant abnormal 12-lead ECG at screening, as deemed by the investigator.
30	The participant is actively enrolled in another clinical study.
31	The participant is prone to keloid or hypertrophic scar formation or any wound healing disorder.
32	Tattoos, or broken skin (other than AD damaged skin) at the insertion areas.
33	Needle phobia.

Table S2 – Flow cytometry: Gating strategy for deep immunophenotyping (surface markers)

**Reported marker**	**As % of**	**Phenotype**	**Gating: initially gating cells, single cells and CD45+ cells**
% CD3+ T cells	CD45+	CD45+CD3+	(i) Display CD3 histogram, on CD3+
% CD4+T cells	CD45+CD3+	CD45+CD3+CD4+CD8-	(i) Display CD3 histogram, on CD3+, (ii) CD4 vs. CD8, on CD4+CD8-
% CD8+ T cells	CD45+CD3+	CD45+CD3+CD4-CD8+	(i) Display CD3 histogram, on CD3+, (ii) CD4 vs. CD8, on CD4-CD8+
% Tregs	CD4+	CD45+CD3+CD4+CD8-CD25+CD127low	(i) Display CD3 histogram, on CD3+, (ii) CD4 vs. CD8, on CD4+CD8-, (iii) CD127 vs. CD25, on CD25+CD127-/low
% Monocytes	CD45+	CD45+CD14+SSClow	(i) Display CD14 vs. SSC-A, on CD14+SSC-A low
% CD3-	CD45+	CD45+CD3-	(i) Display CD3 histogram, on CD3-
% NK cells	CD45+CD3-	CD45+CD3-CD16+CD56+	(i) Display CD3 histogram, on CD3-, (ii) CD16 vs. CD56, on CD16+CD56+
% neutrophil granulocytes	CD45+	CD45+CD16+CD15++	(i) Display CD16 vs. CD15, on CD16+CD15high
% eosinophil granulocytes	CD45+	CD45+CD16mediumCD15+	(i) Display CD16 vs. CD15, on CD16medCD15+
% CD15+	CD45+	CD45+CD15+	(i) Display CD15 histogram, on CD15+
% NKT cells	CD45+	CD45+CD3+CD56+	(i) Display CD3 vs. CD56, on CD3+CD56+
% B cells	CD45+	CD45+CD3-CD19+	(i) Display CD3 vs. CD19, on CD3-CD19+
% SSClowCD45dim	CD45+	CD45+SSClowCD45dim	(i) Display CD45 vs. SSC-A, on CD45-/dimSSC-A-
% CD203c+ basophil	SSClowCD45dim	CD45+SSClowCD45dimCD203c+CD123-	(i) Display CD45 vs. SSC-A, on CD45-/dimSSC-A-, (ii) CD203c vs. CD123, on CD203cposCD123-/dim

Table S3 – Flow cytometry: Gating strategy for cytokine producing cells (intracellular markers)

Reported marker	As % of	Phenotype	Gating: initially gating cells, single cells and CD45+ lymphocytes
% CD3+	lymphocytes	CD45+CD3+	(i) Display CD3 histogram, on CD3+
% CD8+	CD3+ T cells	CD45+CD3+CD4-CD8+	(i) Display CD3 histogram, on CD3+, (ii) CD4 vs. CD8, on CD4-CD8+
% CD8+IFNgamma+	CD8+ T cells	CD45+CD3+CD4-CD8+IFNgamma+	(i) Display CD3 histogram, on CD3+, (ii) CD4 vs. CD8, on CD4-CD8+, (iii) IFNgamma vs. SSC-A, on IFNgamma+
% CD4+	CD3+ T cells	CD45+CD3+CD4+CD8-	(i) Display CD3 histogram, on CD3+, (ii) CD4 vs. CD8, on CD4+CD8-
% CD4+IFNgamma+	CD4+ T cells	CD45+CD3+CD4+CD8-IFNgamma+	(i) Display CD3 histogram, on CD3+, (ii) CD4 vs. CD8, on CD4+CD8-, (iii) IFNgamma vs. SSC-A, on IFNgamma+
% CD4+CD8+	CD3+ T cells	CD45+CD3+CD4+CD8+	(i) Display CD3 histogram, on CD3+, (ii) CD4 vs. CD8, on CD4+CD8+
% CD4-CD8-	CD3+ T cells	CD45+CD3+CD4-CD8-	(i) Display CD3 histogram, on CD3+, (ii) CD4 vs. CD8, on CD4-CD8-
% CD4+IL5+	CD4+ T cells	CD45+CD3+CD4+CD8-IL5+	(i) Display CD3 histogram, on CD3+, (ii) CD4 vs. CD8, on CD4+CD8-, (iii) IL5 vs. SSC-A, on IL5+
% CD4+IL22+	CD4+ T cells	CD45+CD3+CD4+CD8-IL22+	(i) Display CD3 histogram, on CD3+, (ii) CD4 vs. CD8, on CD4+CD8-, (iii) IL22 vs. SSC-A, on IL22+
% CD4+IL31+	CD4+ T cells	CD45+CD3+CD4+CD8-IL31+	(i) Display CD3 histogram, on CD3+, (ii) CD4 vs. CD8, on CD4+CD8-, (iii) IL31 vs. SSC-A, on IL31+
% CD4+IL13+	CD4+ T cells	CD45+CD3+CD4+CD8-IL13+	(i) Display CD3 histogram, on CD3+, (ii) CD4 vs. CD8, on CD4+CD8-, (iii) IL13 vs. SSC-A, on IL13+
% CD4+IL4+	CD4+ T cells	CD45+CD3+CD4+CD8-IL4+	(i) Display CD3 histogram, on CD3+, (ii) CD4 vs. CD8, on CD4+CD8-, (iii) IL4 vs. SSC-A, on IL4+
% CD4+IL17+	CD4+ T cells	CD45+CD3+CD4+CD8-IL17+	(i) Display CD3 histogram, on CD3+, (ii) CD4 vs. CD8, on CD4+CD8-, (iii) IL17 vs. SSC-A, on IL17+

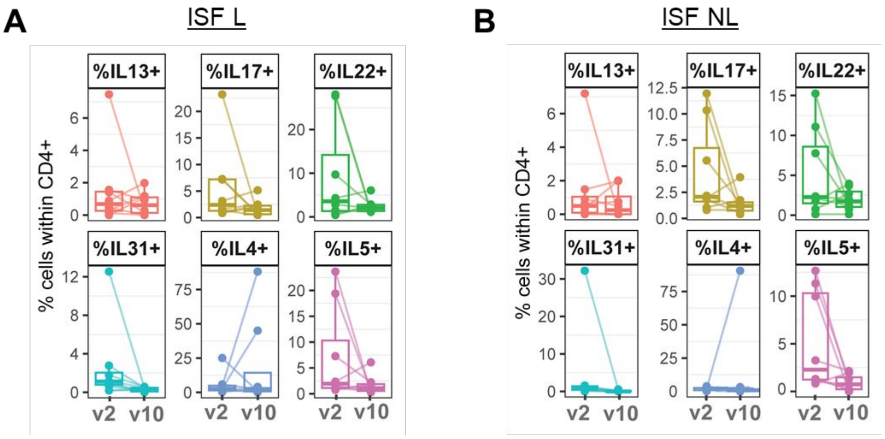

Figure S1 – Parallel coordinates charts showing changes in the populations of CD4+-derived interleukins at baseline (v2) and at the end of treatment (v10) in (A) ISF L and (B) ISF NL.

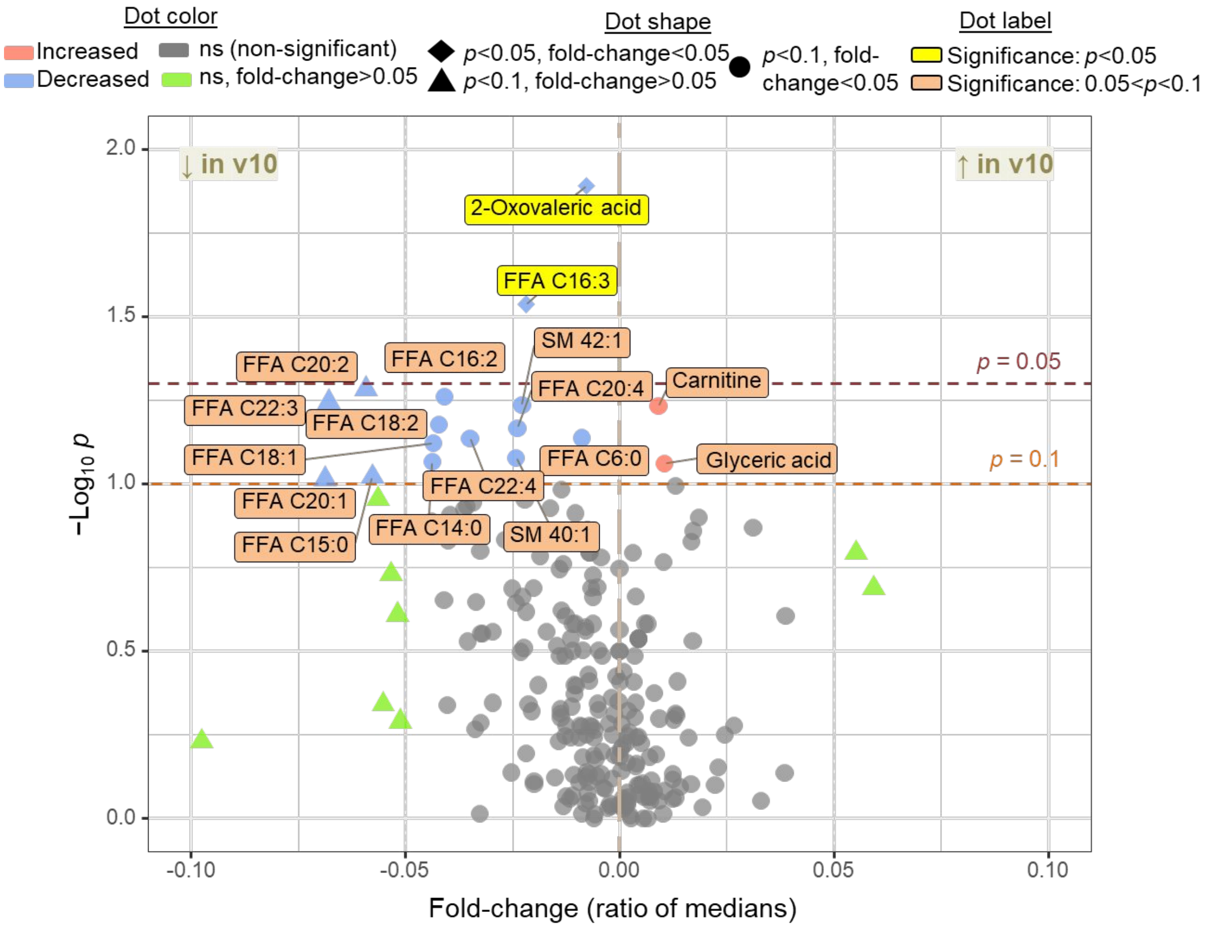

Figure S2 – Volcano plot representing metabolite changes in plasma samples at v10 in relation to baseline (v2). The fold-change was calculated as the ratio of median at v10 minus median at baseline to median at baseline.
